# Ureteroscopy and lasertripsy for lower pole stones <2 cm, *in situ* vs displacement? A systematic review and meta‐analysis

**DOI:** 10.1111/bju.16534

**Published:** 2024-10-13

**Authors:** Arran Dingwall, James Leighton, Angus Luk, Mark Chambers, Bhaskar Somani, Robert Geraghty

**Affiliations:** ^1^ Department of Urology Freeman Hospital Newcastle upon Tyne UK; ^2^ Library Services Newcastle Upon Tyne Hospitals NHS Foundation Trust Newcastle upon Tyne UK; ^3^ Department of Urology University Hospital Southampton Southampton UK; ^4^ Biosciences Institute Newcastle University, Centre for Life Newcastle upon Tyne UK

**Keywords:** ureteroscopy, lower pole, nephrolithiasis, lasertripsy, outcomes, *in‐situ*, displacement

## Abstract

**Objective:**

To investigate the outcomes of ureteroscopy and lasertripsy in lower pole renal stones <2 cm when treated *in situ* compared to displacement to the upper pole.

**Patients and Methods:**

Using the Medical Literature Analysis and Retrieval System Online (MEDLINE)/PubMed, the Excerpta Medica dataBASE (EMBASE), Cumulative Index to Nursing and Allied Health Literature (CINAHL), the Cochrane Library, and Clinicaltrials.gov we identified adult population, English language, studies published until March 2023 comparing surgical outcomes and stone‐free rates (SFRs) in relation to lower pole stones <2 cm managed *in situ* vs those displaced (International Prospective Register of Systematic Reviews [PROSPERO] identifier: CRD42023432750). Analysis was performed using R with the ‘meta’ package. Bias analysis was performed using the Cochrane Risk of Bias 2 tool for randomised trials and the Newcastle–Ottawa scale for observational studies. The Grading of Recommendations, Assessment, Development, and Evaluation (GRADE) approach was used to ascertain the certainty of evidence.

**Results:**

A total of five studies were included, comprising two retrospective cohort studies, three randomised trials, with a total of 408 patients. Meta‐analysis demonstrated SFRs are significantly higher in those patients undergoing displacement vs those managed *in situ* (risk ratio 1.21, 95% confidence interval [CI] 1.10–1.34, *P* < 0.001). There was no significant difference in complication rates. Operative time was significantly longer in the displacement group (mean difference 5.62 min, 95% CI 0.40–10.83 min; *P* = 0.03). Overall risk of bias was moderate. Certainty of evidence was moderate for stone‐free status, and very low for all other outcomes.

**Conclusions:**

This systematic review and meta‐analysis demonstrates that for lower pole stones <2 cm displacement strategies have significantly higher SFRs than treatment *in situ*, with no significant difference in complications. There is significantly increased operative time in the displaced group, but an additional 6 min is unlikely to be clinically significant.

**Abbreviations**
EAU: European Association of Urology
ESWL: extracorporeal shockwave lithotripsy
FURS: flexible ureteroscopy
GRADE: Grading of Recommendations, Assessment, Development, and Evaluation
IPA: infundibulopelvic angle
IPW: infundibulopelvic width
LPS: lower pole stones
NOS: Newcastle–Ottawa Scale
PCNL: percutaneous nephrolithotomy
PICO: Population, Intervention, Comparison and Outcome (framework)
PRISMA: Preferred Reporting Items for Systematic Reviews and Meta‐Analyses
PROSPERO: International Prospective Register of Systematic Reviews
RoB 2: Risk of Bias 2 (tool)
RR: risk ratio
SFR: stone‐free rate
URSL: ureteroscopy and laser lithotripsy

## Introduction

Renal stones have both individual and societal impacts with a significant morbidity [1] and high economic costs [2, 3, 4]. Recent data from the National Health and Nutrition Examination Survey (NHANES) suggests further rises in the prevalence of renal stones in the USA (now 11%), which is likely to be reflective of the developed world [5, 6]. There are three common treatment modalities available for management of renal stones <2 cm: extracorporeal shockwave lithotripsy (ESWL), flexible ureteroscopy (FURS) and percutaneous nephrolithotomy (PCNL), although the AUA [7] and European Association of Urology (EAU) guidelines suggests ESWL or FURS for stones <2 cm [8]. The use of FURS for renal stone treatment has been increasing over the past two decades and is now the predominant treatment type [9]. Lower pole stones (LPS) pose a particular challenge during FURS due to the anatomical and technical complexity of accessing the lower pole [10, 11].

Studies show that these LPS as having lower stone‐free rates (SFRs) during FURS, as low as 60.4% vs up to 94.4% in those stones found in the renal pelvis or upper pole [12, 13, 14]. There are multiple contributing factors to this unfavourable anatomy, including the infundibulopelvic angle (IPA), infundibulopelvic width (IPW), and the length of the inferior calyceal infundibulum [15]. These make FURS more difficult due to the maximum deflection offered by ureteroscopes [10, 11], paired with more difficult anatomy to navigate for the operator [12, 15].

One potential approach to the management of LPS is displacement of stones to the upper renal pole, where these aforementioned complexities are overcome due to the favourable anatomy of the upper pole [13]. We therefore aimed to perform a systematic review and meta‐analysis of displacement of LPS during FURS to the upper pole compared to treatment *in situ*. Our primary outcome was the SFR, with secondary outcomes of complication rate, postoperative stent rate, and operative time.

## Materials and Methods

We report our findings as per the Preferred Reporting Items for Systematic Reviews and Meta‐Analyses (PRISMA) guidelines [16] (Appendix S1).

### Literature Search and Population, Intervention, Comparison and Outcome (PICO) framework

A literature search was carried out by a professional librarian (M.C.), using the Medical Literature Analysis and Retrieval System Online (MEDLINE)/PubMed, the Excerpta Medica dataBASE (EMBASE), Cumulative Index to Nursing and Allied Health Literature (CINAHL) and Cochrane Library. Registered randomised controlled trials (RCTs) were searched using clinicaltrials.gov. The results of this search were reviewed as full‐text manuscripts by two separate authors (A.D. and R.G.).

This literature search was carried out as pre‐defined in the initial protocol on the International Prospective Register of Systematic Reviews (PROSPERO; identifier: CRD42023432750). Study designs included in the search included comparative studies, RCTs and prospective cohorts, as well as retrospective observational studies. Full search terms are summarised in our protocol on PROSPERO. The PICO for this study is detailed below:

Population: all English language, adult patient (aged >18 years) studies.

Intervention and Comparison: studies on ureteroscopy and laser lithotripsy (URSL) for LPS with data of management using *in situ* lasertripsy (intervention) vs displacement and lasertripsy (comparison) for stones <2 cm.

Outcomes: the primary outcome assessed was study defined SFRs (ideally no fragments), with secondary outcomes comprising of operative time, requirement of adjuvant treatments including stenting, and complication rates (ideally graded using the Clavien–Dindo classification [17]).

Exclusion criteria included any case reports or animal studies.

### Data Collection

Data were extracted using a pre‐defined Excel spreadsheet using the criteria above. Sections included: study characteristics of author, country, year of publication and study design; patient and stone demographics of age and sex, stone number, size and burden; and finally operative outcomes including stone‐free status, operative time, need for stenting and perioperative complications. Multiplane measurements of stone burden were converted into single plane by either square or cube root. For outcome measures where only median and interquartile range were supplied, the mean and SDs were calculated as per Wan et al. [18].

### Risk of Bias and Publication Assessment

Risk of bias was assessed with: the Cochrane risk‐of‐bias assessment tool for RCTs [19], and the Newcastle–Ottawa Scale (NOS) of risk of bias for observational studies [20].

Publication bias was assessed via visual inspection of Funnel plots and calculation of Cochran's *Q*. Trim‐and‐fill adjustment was also performed to assess for publication bias, if the number of studies available for analysis was more than three.

### Statistical Analysis

A meta‐analysis was performed using a random‐effect model when heterogeneity was >50%, while the fixed‐effect model was used for heterogeneity <50%. Heterogeneity was assessed using *I* ^2^, tau^2^, and Cochran's *Q*. All statistical analyses were performed using R (R Statistical software, Vienna, Austria) with the ‘meta’ package [21]. As above, we performed trim‐and‐fill analyses to statistically assess publication bias. All pooled summary statistics are resultant from meta‐analyses of proportion (for binary variables) or single continuous variable. Complete analyses along with statistical code is available in Appendix S2. We performed sensitivity analyses based on study design, stone‐free definition and stone size 1–2 cm.

## Results

### Study/Patient Demographics

**Fig. 1:**
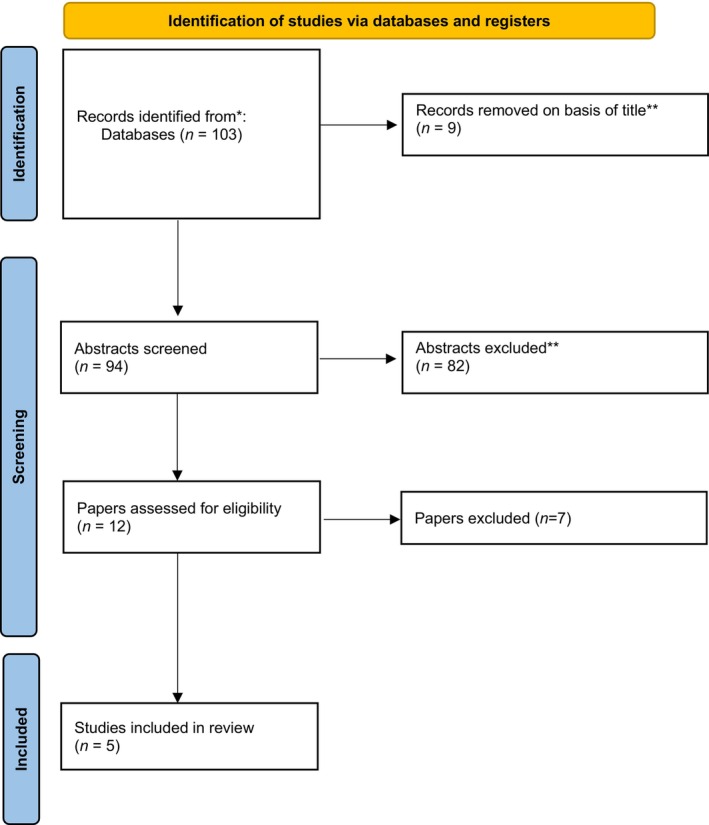
The PRISMA flow diagram for study selection.

Five studies were identified as meeting the inclusion criteria for meta‐analysis [13, 22, 23, 24, 25] (Fig. 1). These studies comprised a total of 408 patients (male, 237; female, 171) and originated from the USA, Nepal and China (Table 1). There were 232 patients in the *in situ* group, and 176 in the displacement group, all stones were <2 cm, with all studies bar Yaghoubian et al. [13]. examining stones 1–2 cm (Table 1) [13, 22, 23, 24, 25].

**Table 1:** Combined patient demographics and study design.

Reference	Country	Study type	Total patients, *N*	Female, *n*	Male, *n*	*In situ* patients, *n*	Displacement patients, *n*	*In situ* group age, years, mean (SD) or median (IQR)	Displacement group age, years, mean (SD) or median (IQR)	*In situ* group stone size, cm, mean (SD) or median (IQR)	Displacement group stone size, cm, mean (SD) or median (IQR)	Laser used	Stone‐free definition
Kourambas et al. [23]	USA	Retrospective cohort	34	12	22	24	10	44	44	1–2	1–2	Ho:YAG	Completely stone free or <02 cm fragments on either non‐contrast CT or IVU at 3 months postoperatively
Schuster et al. [24]	USA	Retrospective cohort	78	34	44	59	19	47	53	0.8	1	Ho:YAG	Completely stone free on plain‐film X‐ray immediately postoperatively
Yaghoubian et al. [13]	USA	RCT	138	69	69	69	69	58 (47–68)	57 (51–64)	0.6 (0.5–0.7)	0.6 (0.2–0.7)	Ho:YAG	Completely stone free on plain‐film X‐ray and US KUB at 30 days postoperatively
Shrestha et al. [25]	Nepal	Prospective randomised study	68	20	48	35	33	33	42	1.2 (0.3)	1.2 (0.3)	Ho:YAG	Completely stone free on X‐ray and US KUB at 4 weeks postoperatively
Huang et al. [22]	China	Prospective randomised study	90	36	54	45	45	53	55	1–2	1–2	Ho:YAG	Completely stone free on X‐ray KUB and <0.2 cm residual stones on US KUB at 3 months postoperatively

KUB, kidneys, ureters and bladder; US, ultrasonography.

All studies utilised a holmium: yttrium‐aluminium‐garnet laser as detailed in Table 1. Use of ureteric access sheaths (UAS) were routine in two studies [22, 25] and surgeon dependent in another [13]. In the remaining two studies [23, 24], UAS were not used; however, if the ureter was found to be unfavourable for ureteroscope passage, balloon dilatation was performed.

In all of these studies nitinol retrieval devices were used, either as a tipless basket or through a nitinol grasper (Table 1). All studies reported complete success of displacement using these devices, with two studies requiring initial *in situ* laser fragmentation of larger stones (sizes of these stones is not provided in the literature) prior to displacement to the upper‐pole [22, 25].

Studies defined stone free as no fragments, with two studies also including <0.2 cm fragments [22, 23]. Three studies reported that no follow‐up procedures were required in patients who were not stone free [13, 22, 23], one study had a single *in situ* patient requiring repeat FURS, with the remaining study having a mixture of repeated FURS, ESWL or PCNL (they did not report on the individual groups these repeated procedures belonged to) [24].

### Meta‐Analysis for Primary Outcome: SFRs in *In Situ* vs Displacement

**Fig. 2:**
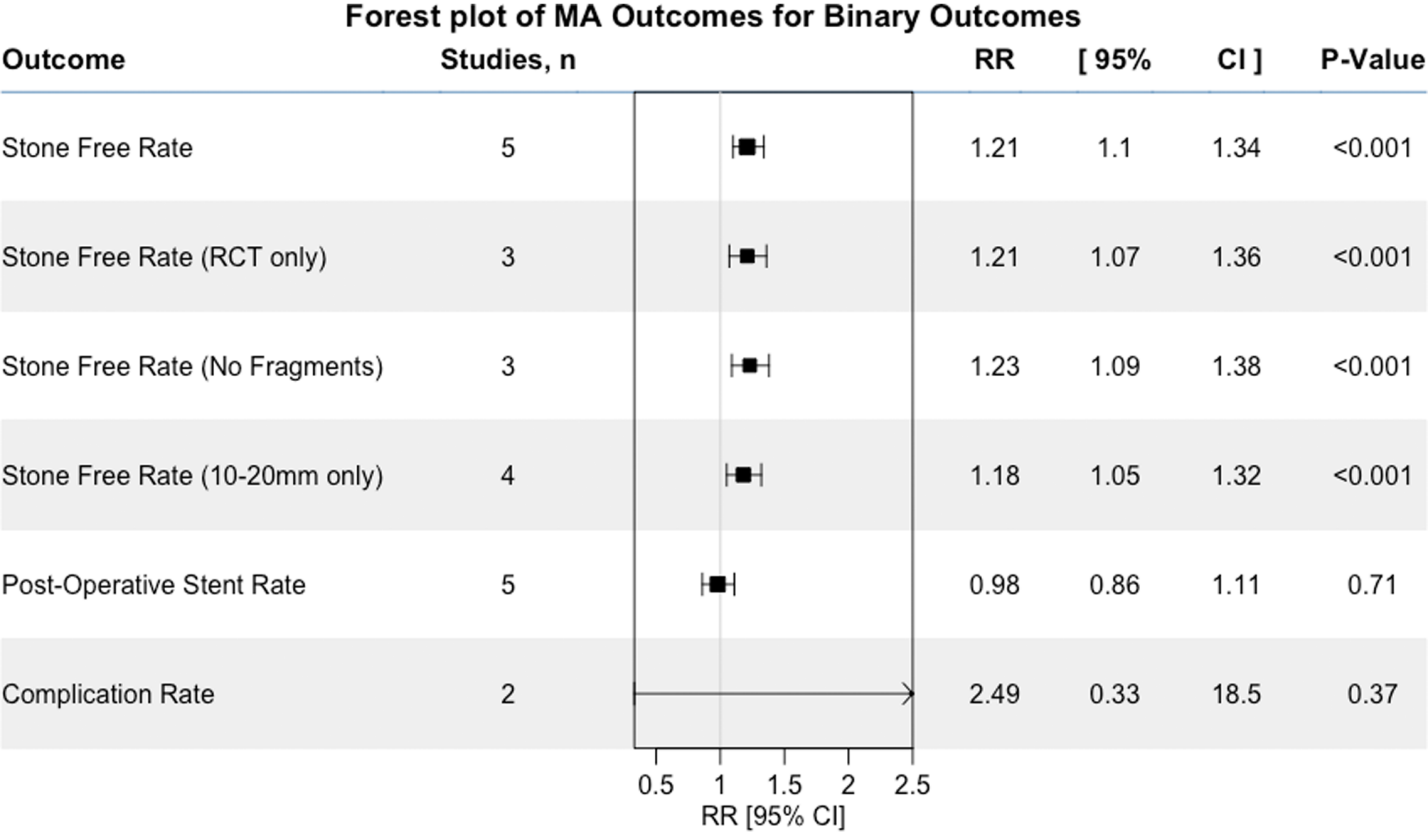
Forest plot summarising all primary and secondary outcomes.

All five studies reported SFRs [13, 22, 23, 24, 25]. On meta‐analysis the SFRs in the displacement group (86%, 95% CI 80–90%) were significantly higher than the *in situ* group (71%, 95% CI 65–77%; Table 2) [13, 22, 23, 24, 25], using the fixed‐effects model (risk ratio [RR] 1.21, 95% CI 1.10–1.34; *P* < 0.001; Figs 2 and S1; Section 3.3: Appendix S2).

**Table 2:** Study outcomes.

Reference	SFR, %		Operative time, min, mean (SD)		Requirement for postoperative stenting, %		Complication rate, %	
	*In situ* group	Displacement group	*In situ* group	Displacement group	*In situ* group	Displacement group	*In situ* group	Displacement group
Kourambas et al. [23]	83	90	49 (no split given)		100	100	4	0
Schuster et al. [24]	71	94	64	80	64	42	12	21
Yaghoubian et al. [13]	74	95	57 (14)*	66 (10)*	65	70	6	12
Shrestha et al. [25]	86	91	43 (14)	48 (13)	100	100	14	18
Huang et al. [22]	67	82	40 (17)	39 (23)	100	100	9	11
Overall (pooled result from meta‐analysis)	72	86	47 (11)	51 (15	76	70	10	14

* Calculated mean from median.

Results showed minimal heterogeneity with *I* ^2^ of 3% and tau^2^ analysis of 0.002. Therefore we opted to use the common effect model for the above outcomes. There was no risk of publication bias found on trim‐and‐fill analysis with *n* = 0 added studies (trim‐and‐fill funnel plot in Fig. S2; Baujat plot in Fig. S5).

Sensitivity analyses of randomised studies only, stone free definition of no fragments only and stones 1–2 cm only, all demonstrated similarly significant results (Fig. 2 and Sections 3.4–3.6: Appendix S2).

### Meta‐Analysis of Postoperative Need for Stenting

Stent rates were reported in all studies [13, 22, 23, 24, 25], some studies routinely stented postoperatively (Table 2), with preoperative stenting also varying between studies (Table 1). There was no significant difference found on meta‐analysis of postoperative stenting as shown in Fig. S3 (RR 0.98, 95% CI 0.86–1.11; *P* = 0.71; see Section 4: Appendix S2). Heterogeneity was again minimal with *I* ^2^ of 0% and tau^2^ of zero so the fixed‐effect model was used.

### Meta‐Analysis of Postoperative Complications

All five studies reported complication rates [13, 22, 23, 24, 25]. There were no significant differences in complication rates between the *in situ* (10%, 95% CI 6–14%) and displacement groups (14%, 95% CI 9–20%) on meta‐analysis (RR 1.51, 95% CI 0.86–2.66; *P* = 0.15; Table 2, Figs 2 and S4; Section 5.1: Appendix S2). The common effect model was again used with *I* ^2^ of 0% and tau^2^ of zero.

Clavien–Dindo grading was available for four studies [13, 22, 23, 25]. Cumulatively by group there were the following numbers of complications: Grade I, *in situ* seven (4%) and displacement five (3%); Grade II, *in situ* six (3%) and displacement 10 (6%); Grade III, *in situ* one (0.6%) and displacement three (2%); Grade IV, *in situ* none and displacement one (0.6%). Pooling Grades I and II showed no significant differences (RR 1.24, 95% CI 0.60–0.26; *P* = 0.55; Section 5.2: Appendix S2). Pooling Grades III and IV also showed no significant differences (RR 2.49, 95% CI 0.33–18.49; *P* = 0.37; Section 5.3: Appendix S2).

### Meta‐Analysis of Operative Time

Three studies reported on operative time [13, 22, 23, 25]. There were significantly longer operative times in the displacement group (pooled mean [SD] 51 [15] min) compared to the *in situ* group (pooled mean [SD] 47 [11] min) on meta‐analysis (mean difference 5.62 min, 95% CI 0.40–10.83 min; *P* = 0.03). Heterogeneity was significant with *I* ^2^ of 55% and tau^2^ of 11.7. Cochran's *Q* was 4.44 (*P* = 0.11). See Section 6: Appendix S2 for further detail.

### Bias Assessment

As above, there was no significant evidence of publication bias found on trim‐and‐fill analysis for our primary outcome of SFRs. For all secondary outcomes there was no evidence of bias and trim and fill was not required to add any results.

For the prospective studies used [13, 22, 25] the Cochrane Risk of Bias 2 (RoB 2) tool [26] was used to assess for risk of bias. We identified low risk of bias in all domains for these first two studies, with some risk of bias identified for Huang et al. [22], as shown in Table 3 [13, 22, 23, 24, 25].

**Table 3:** Bias analysis (green = low risk, amber = some risk, red = high risk).

NOS of risk of bias for observational studies									
Reference	Domains								
	Selection				Comparability	Outcome			
	Representativeness of exposed cohort	Selection of non‐exposed cohort	Ascertainment of exposure	Demonstration that outcome of interest was not present at start of study	Comparability of cohorts	Assessment of outcome	Length of follow‐up	Adequacy of follow‐up cohorts	Overall score (/9)
Kourambas et al. [23]	♦	♦	♦	♦			♦	♦	6
Schuster et al. [24]		♦	♦			♦	♦	♦	5

Cochrane RoB 2 tool for bias analysis in prospective studies						
Reference	Domains					
	Domain 1: risk of bias arising from the randomisation process	Domain 2: risk of bias due to deviations from the intended interventions (effect of assignment to intervention)	Domain 3: missing outcome data	Domain 4: risk of bias in measurement of the outcome	Domain 5: risk of bias in selection of the reported result	Overall risk of bias
Yaghoubian et al. [13]						Low
Shrestha et al. [25]						Low
Huang et al. [22]						Some

The remaining two studies [23, 24] were retrospective observational studies, assessed using the NOS [20]. We identified an outcome of 6 and 5 out of 9 (some concerns for a risk of bias) for each of these respectively.

### Grading of Recommendations, Assessment, Development, and Evaluation (GRADE) Assessment

Table 4 details the GRADE assessment. Given the involvement of non‐randomised studies, this downgraded the evidence. However, for our primary outcome of SFR, we graded the certainty of evidence as moderate. With all other outcomes being very low.

**Table 4:** The GRADE summary assessment.

Certainty assessment							Patients, *n/N* (%)		Effect		Certainty
No. of studies	Study design	Risk of bias	Inconsistency	Indirectness	Imprecision	Other considerations	*In situ* group	Displacement group	Relative (95% CI)	Absolute (95% CI)	
SFR (follow‐up: median 3 months; assessed with: %)											
5	Non‐randomised studies	Not serious	Not serious	Not serious	Serious*	None	168/232 (72.4)	153/176 (86.9)	**RR 1.21** (1.10–1.34)	**183 more per 1000** (from 87 more to 296 more)	⨁◯◯◯ Very low
SFR RCT only (follow‐up: median 3 months)											
3	Randomised trials	Not serious	Not serious	Not serious	Serious*	None	106/149 (71.1)	126/147 (85.7)	RR 1.21 (1.07–1.36)	180 more per 1000 (from 60 more to 309 more)	⨁⨁⨁◯ Moderate
Complications (follow‐up: median 3 months)											
5	Non‐randomised studies	Not serious	Not serious	Not serious	Serious^†^	None	16/176 (9.1)	16/232 (6.9)	RR 1.51 (0.86–2.66)	35 more per 1000 (from 10 fewer to 114 more)	⨁◯◯◯ Very low
Operative time (follow‐up: median 1 days; assessed with: MD)											
3	Non‐randomised studies	Not serious	Not serious	Not serious	Serious*	Publication bias strongly suspected^‡^	102	104	–	MD 5.62 min higher (0.4 higher to 10.83 higher)	⨁◯◯◯ Very low

MD, mean difference.

* Wide CIs.

^†^ Outlier – Huang et al. (2023) [22].

^‡^ Only two studies – cannot make any further assessments statistically.

## Discussion

### Data Summary

This systematic review and meta‐analysis of five studies [13, 22, 23, 24, 25] provides evidence for the use of displacement in the management of LPS, showing a significant increase in SFRs in displaced LPS compared to those managed *in situ*. This is consistent with data from all identified studies showing this same significant increase in SFRs when such stones are displaced [13, 22, 23, 24, 25]. We also demonstrate no significant difference in postoperative stent or complication rates. However, there was a significantly increased operative time in the displacement group compared to the *in situ* group, with a mean difference of ~6 min.

### Strengths of this Systematic‐Review and Meta‐Analysis

The five studies analysed in this systematic review show some bias, with both of the observational studies showing ‘some concern’ of bias from the NOS. This risk of bias comes from neither of these studies accounting for confounding variables; however, reassuringly they both have blindly randomised groups minimising the risk this has to the assessed outcomes. Similarly, data for the prospective study by Huang et al. [22] also showed ‘some bias’ through RoB 2 analysis, due to details on the randomisation process not being present.

Data were also strong regarding heterogeneity, with minimal heterogeneity identified in SFRs, need for stenting and complication rates, with us therefore using common‐effect models throughout. Our sensitivity analyses for our primary outcome (stone‐free status) showed a conserved effect for displacement, which is reflected in the GRADE summary of evidence (moderate).

For the secondary outcomes, there were sufficient studies for meta‐analysis. Of note, four studies reported complications according to the Clavien–Dindo classification, and all studies reported an overall complication rate. Reassuringly, the proportion of complications is low, with only 10% postoperative complications in the *in situ* group and 14% in the displacement group. There were small, non‐significant rises in major complications (Clavien–Dindo Grade ≥III) in the displacement group; however, the sample sizes were likely too small to detect any meaningful difference. This should be a high priority analysis for a subsequent meta‐analysis once further studies have been published.

External validity of this review is also reassuring, with studies having a near‐even split of patients’ sex and with these studies originating from around the globe (China, USA, Nepal), although ideally studies would originate from more than three individual countries.

### Limitations

As shown in the Baujat plot in Fig. S5, we have identified Shrestha et al. [25] as a significant outlier, with over twice the contribution to overall heterogeneity vs the other studies. However, in a leave‐one‐out analysis, the SFRs for displaced stones remained significantly higher than for those left *in situ*.

Another limitation is shared with many similar aspects of FURS research, and there are confounding variables arising from both study design and variations in patient anatomy. Previous studies have shown that success of FURS may also be related to renal tract anatomy including the infundibulopelvic length, the IPW and IPA [27]. As none of the studies assessed in this review included these data, we are unable to comment on if these have acted as confounding variables. However, the sensitivity analysis of randomised trials, in which anatomical complexity should be evenly distributed across the two groups, do suggest that the treatment effect is conserved.

These study design variables included different methods of identification of stone‐free status through each study. All except one study identified stone‐free status as ‘complete’, with them utilising a range of imaging from ultrasonography and CT to X‐ray. They all differed in when this imaging was used to identify stone‐free status, ranging from immediately postoperatively to 3 months (Table 1). Furthermore, not all studies identified preoperative stone size or burden, giving a significant unaccounted for in this variable.

We identified a statistically significant increase in operative time for the displacement group vs *in situ* group. However, an ~6 min increase is unlikely to be clinically significant, with the total mean duration in this group of 51 min, remaining under the 90 min recommended as the cut‐off by the EAU guidelines [28].

### Possible Areas of Future Research

As discussed above, research suggests that the IPA has a significant bearing on stone‐free status when performing URSL on LPS [15, 27, 29]. Given the significance of the IPA on stone‐free status, future research looking into the IPA with relationship to displacement, ideally identifying cut‐offs where this is most beneficial, would be valuable to this field.

Similar to research on anatomical variations, there is ever‐growing research into variations of stone burden and how that may affect outcomes. The PuRE RCT (International Standard Randomised Controlled Trial Number 98970319; publication pending) has recently been presented at both the AUA and EAU annual meetings, with evidence suggesting that ESWL is the most cost‐effective method of treatment for LPS <1 cm with ‘no meaningful difference in patient health status despite the higher complete stone free rates with FURS’ [5]. They further suggest that PCNL is the most cost‐effective method for treatment of stones 1–2.5 cm [5]. Given these data, it suggests that FURS may become less utilised, although the authors would always advocate for a patient‐centred approach, accounting for individual preferences and contraindications. We eagerly await the published results of the PuRE trial. Further randomised trials into management of LPS with relation to stone burden and anatomical complexity are warranted, as there is currently minimal data on displacement techniques in relation to different stone burdens.

## Conclusion

This study presents the most robust evidence to date of using displacement techniques during FURS in the treatment of LPS. The findings presented in this paper have evidenced how displacement of LPS to the upper renal pole has a significantly increased chance of rendering a patient stone‐free following completion of lasertripsy. There is a significant increase in operative time of ~6 min in the displacement group; however, this is likely to be clinically insignificant. There were no significant differences in complication rates. Further trials are needed to improve the certainty of evidence.

## Disclosure of Interests

Robert Geraghty is funded by the Royal College of Surgeons of England and is an associate member of the EAU Guidelines panel on Urolithiasis. Bhaskar Somani is a member of the EAU Guidelines panel on Urolithiasis.

## Author Contributions

Conceptualisation: Bhaskar Somani, Robert Geraghty; Methodology: Robert Geraghty; Data collection: Mark Chambers, Arran Dingwall, Robert Geraghty; Data curation: Robert Geraghty; Statistical analysis: Robert Geraghty; Writing–original draft: Arran Dingwall, James Leighton; Writing–review and editing: Robert Geraghty, Angus Luk, Bhaskar Somani; Supervision: Robert Geraghty.

## Supporting information


**Appendix S1.** The PRISMA guidelines.

**Appendix S2.** *In situ* vs displacement.

**Figure S1.** Forest plot summarising the primary outcome of SFRs of *in situ* (control group) vs displacement (experimental group) in the management of lower pole stones. ‘Events’ represent number of stone‐free patients in each group.

**Figure S2.** Funnel plot demonstrating trim‐and‐fill analysis for meta‐analysis of SFRs.

**Figure S3.** Forest plot summarising the secondary outcome of perioperative stenting between *in situ* (control group) vs displacement (experimental group).

**Figure S4.** Forest plot summarising the secondary outcome of complication rates between *in situ* (control group) vs displacement (experimental group).

**Figure S5.** Baujat plot of primary outcome—SFRs—demonstrating each studies contribution to heterogeneity.

## References

[1] Tapiero S , Limfuco L , Bechis SK et al. The impact of the number of lifetime stone events on quality of life: results from the north American stone quality of life consortium. Urolithiasis 2021; 49: 321–326 33409555 10.1007/s00240-020-01238-y

[2] Hyams ES , Matlaga BR . Economic impact of urinary stones. Transl Androl Urol 2014; 3: 278–283 26816777 10.3978/j.issn.2223-4683.2014.07.02 PMC4708578

[3] Geraghty RM , Cook P , Walker V , Somani BK . Evaluation of the economic burden of kidney stone disease in the UK: a retrospective cohort study with a mean follow‐up of 19 years. BJU Int 2020; 125: 586–594 31916369 10.1111/bju.14991

[4] Antonelli JA , Maalouf NM , Pearle MS , Lotan Y . Use of the National Health and Nutrition Examination Survey to calculate the impact of obesity and diabetes on cost and prevalence of urolithiasis in 2030. Eur Urol 2014; 66: 724–729 25015037 10.1016/j.eururo.2014.06.036 PMC4227394

[5] McClinton S , Starr K , Thomas R et al. The clinical and cost effectiveness of surgical interventions for stones in the lower pole of the kidney: the percutaneous nephrolithotomy, flexible ureterorenoscopy and extracorporeal shockwave lithotripsy for lower pole kidney stones randomised controlled trial (PUrE RCT) protocol. Trials 2020; 21: 1–15 32498699 10.1186/s13063-020-04326-x PMC7273687

[6] Hill AJ , Basourakos SP , Lewicki P et al. Incidence of kidney stones in the United States: the continuous National Health and Nutrition Examination Survey. J Urol 2022; 207: 851–856 34854755 10.1097/JU.0000000000002331

[7] Assimos D , Krambeck A , Miller NL et al. Surgical management of stones: American urological association/endourological society guideline, Part I. J Urol 2016; 196: 1153–1160 27238616 10.1016/j.juro.2016.05.090

[8] Geraghty RM , Davis NF , Tzelves L et al. Best practice in interventional management of urolithiasis: an update from the European Association of Urology Guidelines Panel for Urolithiasis 2022. Eur Urol Focus 2023; 9: 199–208 35927160 10.1016/j.euf.2022.06.014

[9] Geraghty RM , Jones P , Somani BK . Worldwide trends of urinary stone disease treatment over the last two decades: a systematic review. J Endourol 2017; 31: 547–556 28095709 10.1089/end.2016.0895

[10] Lb D , Bk S , Ex K et al. Characteristics of current digital single‐use flexible ureteroscopes versus their reusable counterparts: an in‐vitro comparative analysis. Transl Androl Urol 2019; 8(Suppl 4): S359–S370 31656742 10.21037/tau.2019.09.17 PMC6790413

[11] Dragos LB , Somani BK , Sener ET et al. Which flexible ureteroscopes (digital vs. fiber‐optic) can easily reach the difficult lower pole calices and have better end‐tip deflection: in vitro study on K‐box. A PETRA evaluation. J Endourol 2017; 31: 630–637 28478744 10.1089/end.2017.0109

[12] Moore SL , Bres‐Niewada E , Cook P , Wells H , Somani BK . Optimal management of lower pole stones: the direction of future travel. Cent Eur J Urol 2016; 69: 274–279 10.5173/ceju.2016.819 PMC5057048 27729994

[13] Yaghoubian A , Anastos H , Khusid J et al. Displacement of lower pole stones during retrograde intrarenal surgery improves stone‐free status: a prospective randomized controlled trial. J Urol 2023; 209: 963–970 36753676 10.1097/JU.0000000000003199

[14] Lim SH , Jeong BC , Seo SI , Jeon SS , Han DH . Treatment outcomes of retrograde intrarenal surgery for renal stones and predictive factors of stone‐free. Korean J Urol 2010; 51: 777–782 21165199 10.4111/kju.2010.51.11.777 PMC2991576

[15] Geavlete P , Multescu R , Geavlete B . Influence of pyelocaliceal anatomy on the success of flexible ureteroscopic approach. J Endourol 2008; 22: 2235–2239 18937587 10.1089/end.2008.9719

[16] Page MJ , McKenzie JE , Bossuyt PM et al. The PRISMA 2020 statement: an updated guideline for reporting systematic reviews. BMJ 2021; 372: n71 33782057 10.1136/bmj.n71 PMC8005924

[17] Dindo D , Demartines N , Clavien PA . Classification of surgical complications: a new proposal with evaluation in a cohort of 6336 patients and results of a survey. Ann Surg 2004; 240: 205–213 15273542 10.1097/01.sla.0000133083.54934.ae PMC1360123

[18] Wan X , Wang W , Liu J , Tong T . Estimating the sample mean and standard deviation from the sample size, median, range and/or interquartile range. BMC Med Res Methodol 2014; 14: 135 25524443 10.1186/1471-2288-14-135 PMC4383202

[19] Higgins JPT , Altman DG , Gøtzsche PC et al. The Cochrane Collaboration's tool for assessing risk of bias in randomised trials. BMJ 2011; 343: d5928 22008217 10.1136/bmj.d5928 PMC3196245

[20] Ottawa Hospital Research Institute [Internet]. Available at: https://www.ohri.ca/programs/clinical_epidemiology/oxford.asp. Accessed May 2024

[21] Balduzzi S , Rücker G , Schwarzer G . How to perform a meta‐analysis with R: a practical tutorial. Evid Based Ment Health 2019; 22: 153–160 31563865 10.1136/ebmental-2019-300117 PMC10231495

[22] Huang R , Chun CJ , Qiang ZY et al. Relocation of lower pole renal stones helps improve the stone‐free rate during flexible ureteroscopy with a low complication rate. World J Urol 2024; 42: 30 38217719 10.1007/s00345-023-04703-6 PMC10787685

[23] Kourambas J , Delvecchio FC , Munver R , Preminger GM . Nitinol stone retrieval‐assisted ureteroscopic management of lower pole renal calculi. Urology 2000; 56: 935–939 11113736 10.1016/s0090-4295(00)00821-9

[24] Schuster TG , Hollenbeck BK , Faerber GJ , Wolf JS Jr . Ureteroscopic treatment of lower pole calculi: comparison of lithotripsy in situ and after displacement. J Urol 2002; 168: 43–45 12050489

[25] Shrestha A , Adhikari B , Shah AK . Does relocation of lower pole stone during retrograde intrarenal surgery improve stone‐free rate? A prospective randomized study. J Endourol 2023; 37: 21–27 36074950 10.1089/end.2022.0050

[26] Sterne JAC , Savović J , Page MJ et al. RoB 2: a revised tool for assessing risk of bias in randomised trials. BMJ 2019; 366: l4898 31462531 10.1136/bmj.l4898

[27] Luk A , Geraghty R , Somani B . Endourological options for small (< 2 cm) lower pole stones — does the lower pole angle matter? Curr Urol Rep 2023; 24: 365–370 37097431 10.1007/s11934-023-01161-w PMC10403423

[28] Uroweb ‐ European Association of Urology [Internet] . EAU Guidelines on Urolithiasis ‐ GUIDELINES – Uroweb. Available at: https://uroweb.org/guidelines/urolithiasis/chapter/guidelines. Accessed May 2024

[29] Kilicarslan H , Kaynak Y , Kordan Y et al. Unfavorable anatomical factors influencing the success of retrograde intrarenal surgery for lower pole renal calculi. Urol J 2015; 12: 2065–2068 25923149

