# Supplementary material for: Ureteroscopy and lasertripsy for lower pole stones <2 cm, in situ vs displacement? A systematic review and meta‐analysis
Source: BJU Int. 2024 Oct 13;135(3):399–407. doi: 10.1111/bju.16534 (PMC11842885; doi:10.1111/bju.16534)

In Situ vs Displacement

R Geraghty

2024-05-07

Contents

1 Setup **2**

2 Data Exploration **3**
2.1 Plot missing data . . . 3

3 Stone Free Status **5**
3.1 Meta-analysis of Proportions for In Situ . . . 6
3.2 Meta-analysis of Proportions for Displacement . . . 7
3.3 Meta-Analysis (in situ (reference) vs displacement) . . . 8
3.3.1 Result . . . 8
3.3.2 Forest plot . . . 9
3.3.3 Trim and Fill . . . 10
3.3.4 Baujat . . . 12
3.3.5 Leave One Out . . . 13
3.4 RCT only Meta-Analysis (in situ (reference) vs displacement) . . . 14
3.4.1 Result . . . 14
3.4.2 Forest plot . . . 15
3.4.3 Trim and Fill . . . 16
3.4.4 Baujat . . . 18
3.4.5 Leave One Out . . . 19
3.5 Stone Free definition Meta-Analysis (in situ (reference) vs displacement) . . . 20
3.5.1 Result . . . 20
3.5.2 Forest plot . . . 21
3.5.3 Trim and Fill . . . 22
3.5.4 Baujat . . . 24
3.5.5 Leave One Out . . . 25
3.6 Stones 10mm-20mm Meta-Analysis (in situ (reference) vs displacement) . . . 26
3.6.1 Result . . . 26
3.6.2 Forest plot . . . 27
3.6.3 Trim and Fill . . . 28
3.6.4 Baujat . . . 30
3.6.5 Leave One Out . . . 31

4 Need for Stenting **32**
4.1 Meta-analysis of Proportions for In Situ . . . 33
4.2 Meta-analysis of Proportions for Displacement . . . 34
4.3 Meta-Analysis . . . 35
4.3.1 Result . . . 35
4.3.2 Forest plot . . . 36
4.3.3 Trim and Fill . . . 37
4.3.4 Baujat . . . 39

4.3.5 Leave One Out . . . 40

5 Complications **41**
5.1 Overall . . . 42
5.1.1 Data Exploration . . . 42
5.1.2 Meta-analysis of Proportions for In Situ . . . 43
5.1.3 Meta-analysis of Proportions for Displacement . . . 44
5.1.4 Overall Meta-Analysis . . . 45
5.1.5 Forest plot . . . 46
5.1.6 Trim and Fill . . . 47
5.1.7 Baujat . . . 49
5.1.8 Leave One Out . . . 50
5.2 Clavien Dindo I-II . . . 51
5.2.1 Meta-analysis of Proportions for In Situ . . . 51
5.2.2 Meta-analysis of Proportions for Displacement . . . 52
5.2.3 Meta-Analysis . . . 53
5.2.4 Forest plot . . . 54
5.2.5 Trim and Fill . . . 55
5.2.6 Baujat . . . 57
5.2.7 Leave One Out . . . 58
5.3 Clavien Dindo III . . . 59
5.3.1 Meta-analysis of Proportions for In Situ . . . 59
5.3.2 Meta-analysis of Proportions for Displacement . . . 60
5.3.3 Meta-Analysis . . . 61
5.3.4 Forest plot . . . 62
5.3.5 Trim and Fill . . . 63
5.3.6 Baujat . . . 64
5.3.7 Leave One Out . . . 65

6 Operative time **66**
6.1 Meta-analysis of individual components . . . 67
6.1.1 In Situ . . . 67
6.1.2 Displacement . . . 68
6.2 Meta-Analysis . . . 69
6.2.1 Result . . . 69
6.2.2 Forest plot . . . 70
6.2.3 Trim and Fill . . . 71
6.2.4 Baujat . . . 73

7 Overall Results **74**
7.0.1 In Situ vs control . . . 74
7.1 Summary Forest Plot . . . 76

1 Setup

2 Data Exploration

2.1 Plot missing data

```
insitu_data$study_type <- as.factor(insitu_data$study_type)
insitu_data$in_situ_stone_free_n <- as.integer(insitu_data$in_situ_stone_free_n)
insitu_data$number_in_situ <- as.integer(insitu_data$number_in_situ)
insitu_data$number_displaced <- as.integer(insitu_data$number_displaced)
insitu_data$displacement_stone_free_n <- as.integer(insitu_data$displacement_stone_free_n)
insitu_data$in_situ_operative_time <- as.numeric(insitu_data$in_situ_operative_time) %>% round(digits =
```

```
## Warning in as.numeric(insitu_data$in_situ_operative_time) %>% round(digits =
## 1): NAs introduced by coercion
```

```
insitu_data$displacement_operative_time <- as.numeric(insitu_data$displacement_operative_time) %>% roun
```

```
## Warning in as.numeric(insitu_data$displacement_operative_time) %>% round(digits
## = 1): NAs introduced by coercion
```

```
insitu_data$in_situ_operative_time_sd <- as.numeric(insitu_data$in_situ_operative_time_sd) %>% round(di
insitu_data$displacement_operative_time_sd <- as.numeric(insitu_data$displacement_operative_time_sd) %>

insitu_data$in_situ_clavien_dindo_1 <- as.integer(insitu_data$in_situ_clavien_dindo_1)
```

```
## Warning: NAs introduced by coercion
```

```
insitu_data$displaced_clavien_dindo_1 <- as.integer(insitu_data$displaced_clavien_dindo_1)
```

```
## Warning: NAs introduced by coercion
```

```
insitu_data$in_situ_clavien_dindo_2 <- as.integer(insitu_data$in_situ_clavien_dindo_2)
```

```
## Warning: NAs introduced by coercion
```

```
insitu_data$displaced_clavien_dindo_2 <- as.integer(insitu_data$displaced_clavien_dindo_2)
```

```
## Warning: NAs introduced by coercion
```

```
insitu_data$in_situ_clavien_dindo_3 <- as.integer(insitu_data$in_situ_clavien_dindo_3)
```

```
## Warning: NAs introduced by coercion
```

```
insitu_data$displaced_clavien_dindo_3 <- as.integer(insitu_data$displaced_clavien_dindo_3)
```

```
## Warning: NAs introduced by coercion
```

```
insitu_data$in_situ_clavien_dindo_4 <- as.integer(insitu_data$in_situ_clavien_dindo_4)
```

```
## Warning: NAs introduced by coercion
```

```
insitu_data$displaced_clavien_dindo_4 <- as.integer(insitu_data$displaced_clavien_dindo_4)
```

```
## Warning: NAs introduced by coercion
```

```
plot_missing(insitu_data)
```

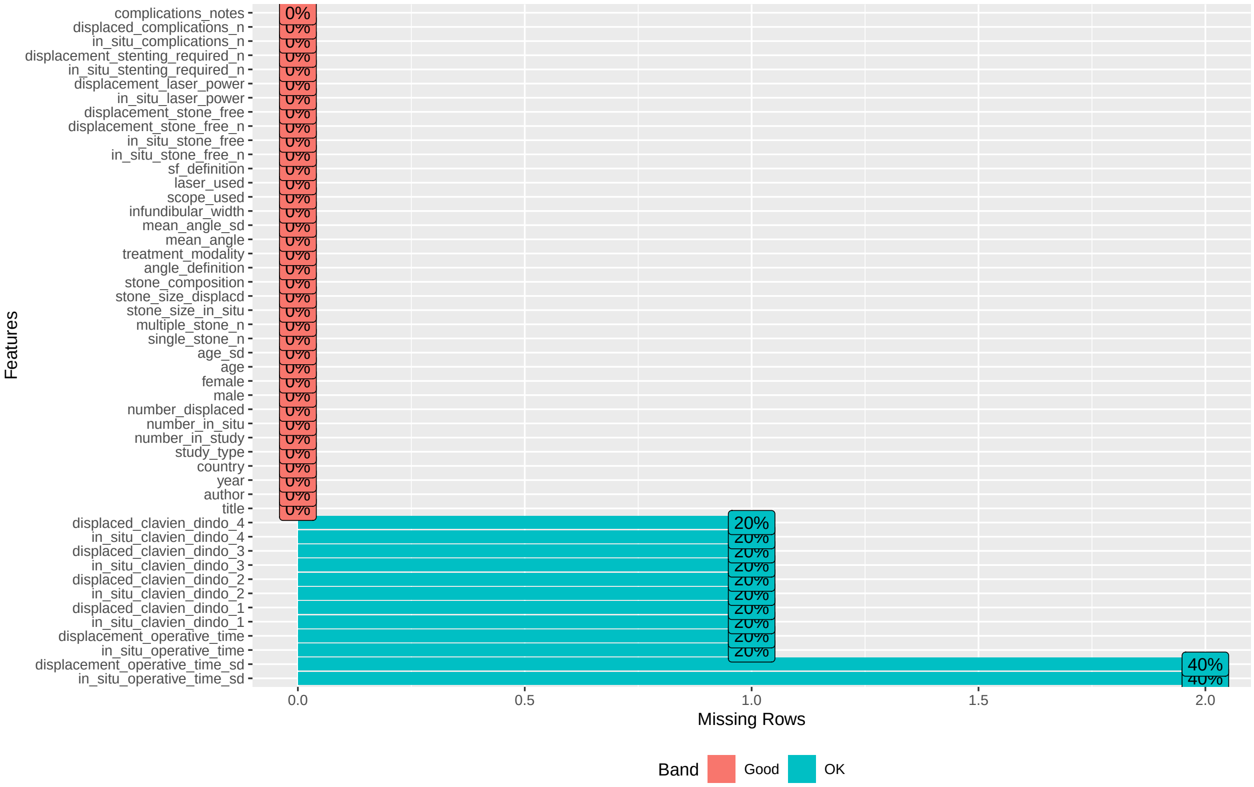

complications_notes
displaced_complications_n
in_situ_complications_n
displacement_stenting_required_n
in_situ_stenting_required_n
displacement_laser_power
in_situ_laser_power
displacement_stone_free
displacement_stone_free_n
in_situ_stone_free
in_situ_stone_free_n
sf_definition
laser_used
scope_used
infundibular_width
mean_angle_sd
mean_angle
treatment_modality
angle_definition
stone_composition
stone_size_displacd
stone_size_in_situ
multiple_stone_n
single_stone_n
age_sd
age
female
male
number_displaced
number_in_situ
number_in_study
study_type
country
year
author
title
displaced_clavien_dindo_4
in_situ_clavien_dindo_4
displaced_clavien_dindo_3
in_situ_clavien_dindo_3
displaced_clavien_dindo_2
in_situ_clavien_dindo_2
displaced_clavien_dindo_1
in_situ_clavien_dindo_1
displacement_operative_time
in_situ_operative_time
displacement_operative_time_sd
in_situ_operative_time_sd
0%
20%
40%
Features
0.0
0.5
1.0
1.5
2.0
Missing Rows
Band
Good
OK

3 Stone Free Status

3.1 Meta-analysis of Proportions for In Situ

```
in_situ_metaprop <- meta::metaprop(
  data = insitu_data,
  event = in_situ_stone_free_n,
  n = number_in_situ,
  studlab = paste(author, year, sep = ", "),
  method = "Inverse"
)
in_situ_metaprop
```

```
## Number of studies: k = 5
## Number of observations: o = 232
## Number of events: e = 168
##
##                        proportion           95%-CI
## Common effect model        0.7155 [0.6525; 0.7710]
## Random effects model       0.7215 [0.6497; 0.7836]
##
## Quantifying heterogeneity:
##  tau^2 = 0.0284 [0.0000; 2.0861]; tau = 0.1686 [0.0000; 1.4443]
##  I^2 = 34.9% [0.0%; 75.5%]; H = 1.24 [1.00; 2.02]
##
## Test of heterogeneity:
##     Q d.f.  p-value
##  6.15    4  0.1884
##
## Details on meta-analytical method:
## - Inverse variance method
## - Restricted maximum-likelihood estimator for tau^2
## - Q-Profile method for confidence interval of tau^2 and tau
## - Logit transformation
```

3.2 Meta-analysis of Proportions for Displacement

```
displacement_metaprop <- metaprop(
  event = displacement_stone_free_n,
  n = number_displaced,
  data = insitu_data,
  studlab = paste(author, year, sep = ", "),
  method = "Inverse"
)
displacement_metaprop
```

```
## Number of studies: k = 5
## Number of observations: o = 176
## Number of events: e = 153
##
##                        proportion           95%-CI
## Common effect model        0.8629 [0.8019; 0.9073]
## Random effects model       0.8629 [0.8019; 0.9073]
##
## Quantifying heterogeneity:
##  tau^2 = 0 [0.0000; 1.6827]; tau = 0 [0.0000; 1.2972]
##  I^2 = 0.0% [0.0%; 79.2%]; H = 1.00 [1.00; 2.19]
##
## Test of heterogeneity:
##     Q d.f. p-value
##  2.41    4  0.6616
##
## Details on meta-analytical method:
## - Inverse variance method
## - Restricted maximum-likelihood estimator for tau^2
## - Q-Profile method for confidence interval of tau^2 and tau
## - Logit transformation
```

3.3 Meta-Analysis (in situ (reference) vs displacement)

3.3.1 Result

```
sf_insitu_rma <- metabin(data = insitu_data,
                   event.c = in_situ_stone_free_n,
                   n.c = number_in_situ,
                   event.e = displacement_stone_free_n,
                   n.e = number_displaced,
                   studlab = paste(author, year, sep = ", ")
                   )
sf_insitu_rma
```

```
## Number of studies: k = 5
## Number of observations: o = 408
## Number of events: e = 321
##
##                          RR           95%-CI    z p-value
## Common effect model  1.2146 [1.0993; 1.3420] 3.82  0.0001
## Random effects model 1.1951 [1.0801; 1.3224] 3.45  0.0006
##
## Quantifying heterogeneity:
##  tau^2 = 0.0020 [0.0000; 0.0754]; tau = 0.0445 [0.0000; 0.2747]
##  I^2 = 2.8% [0.0%; 79.8%]; H = 1.01 [1.00; 2.22]
##
## Test of heterogeneity:
##     Q d.f. p-value
##  4.12    4  0.3905
##
## Details on meta-analytical method:
## - Mantel-Haenszel method
## - Inverse variance method
## - Restricted maximum-likelihood estimator for tau^2
## - Q-Profile method for confidence interval of tau^2 and tau
```

3.3.2 Forest plot

```r
forest(sf_insitu_rma,
       sortvar = TE)
```

Study	Experimental Events	Experimental Total	Control Events	Control Total	RR	95%−CI	Weight (common)	Weight (random)
Shrestha et−al, 2023	30	33	30	35	1.06	[0.89; 1.26]	21.2%	27.3%
Kourambas et−al, 2000	9	10	20	24	1.08	[0.82; 1.42]	8.6%	12.4%
Huang et−al, 2023	37	45	30	45	1.23	[0.96; 1.58]	21.8%	14.9%
Yaghoubian et−al, 2023	59	69	46	69	1.28	[1.06; 1.56]	33.5%	22.8%
Schuster et−al, 2002	18	19	42	59	1.33	[1.10; 1.62]	14.9%	22.6%
**Common effect model**		**176**		**232**	**1.21**	**[1.10; 1.34]**	**100.0%**	**−−**
**Random effects model**					**1.20**	**[1.08; 1.32]**	**−−**	**100.0%**

Heterogeneity: $I^2 = 3\%$, $\tau^2 = 0.0020$, $p = 0.39$

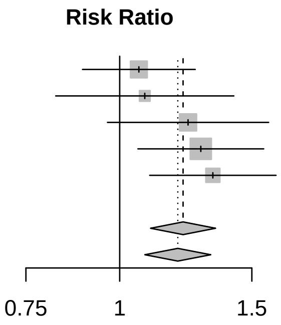

3.3.3 Trim and Fill

```r
trimfill(sf_insitu_rma)
```

```
## Number of studies: k = 5 (with 0 added studies)
## Number of observations: o = 408
## Number of events: e = 321
##
##                          RR           95%-CI    z p-value
## Random effects model 1.1951 [1.0801; 1.3224] 3.45  0.0006
##
## Quantifying heterogeneity:
##  tau^2 = 0.0020 [0.0000; 0.0754]; tau = 0.0445 [0.0000; 0.2747]
##  I^2 = 2.8% [0.0%; 79.8%]; H = 1.01 [1.00; 2.22]
##
## Test of heterogeneity:
##     Q d.f. p-value
##  4.12    4  0.3905
##
## Details on meta-analytical method:
## - Inverse variance method
## - Restricted maximum-likelihood estimator for tau^2
## - Q-Profile method for confidence interval of tau^2 and tau
## - Trim-and-fill method to adjust for funnel plot asymmetry (L-estimator)
```

```r
funnel(trimfill(sf_insitu_rma))
```

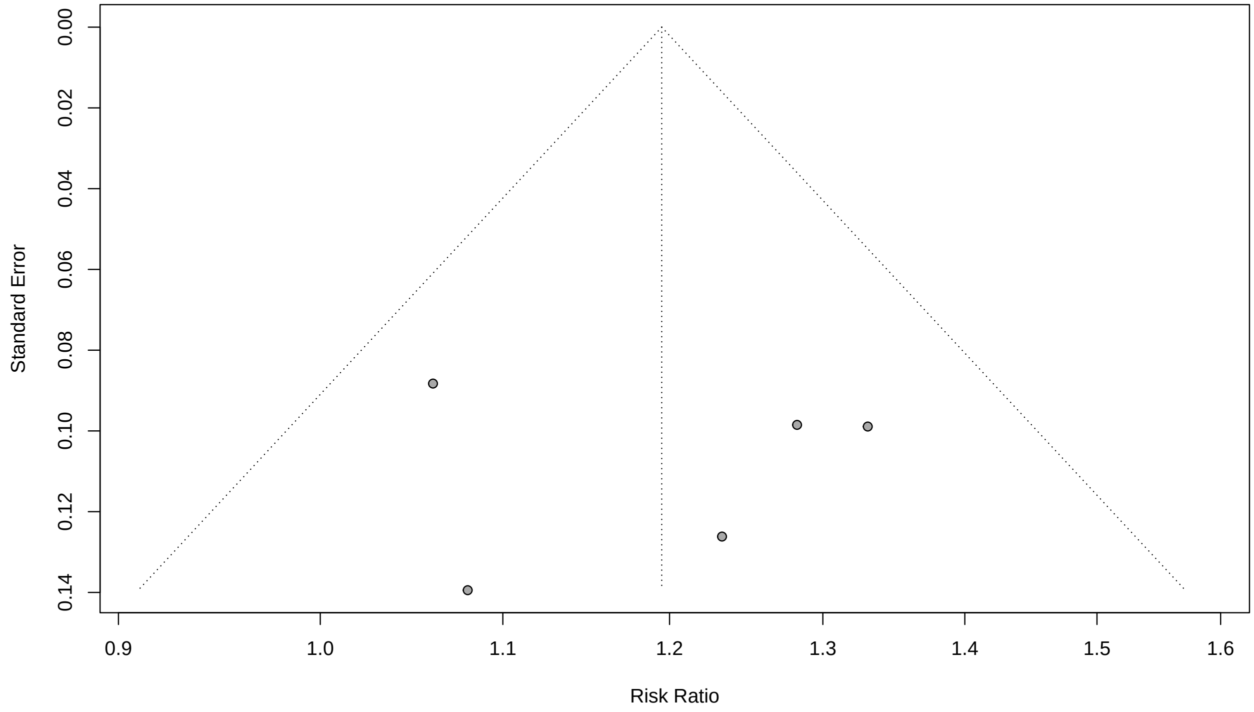


```r
forest(trimfill(sf_insitu_rma), sortvar = TE)
```

Study	logRR	SE(logRR)	Experimental Total	Control Total	Risk Ratio	RR	95%−CI	Weight
Shrestha et−al, 2023	0.0588	0.0883	33	35		1.06	[0.89; 1.26]	27.3%
Kourambas et−al, 2000	0.0770	0.1394	10	24		1.08	[0.82; 1.42]	12.4%
Huang et−al, 2023	0.2097	0.1262	45	45		1.23	[0.96; 1.58]	14.9%
Yaghoubian et−al, 2023	0.2489	0.0985	69	69		1.28	[1.06; 1.56]	22.8%
Schuster et−al, 2002	0.2858	0.0989	19	59		1.33	[1.10; 1.62]	22.6%
**Random effects model**			**176**	**232**		**1.20**	**[1.08; 1.32]**	**100.0%**

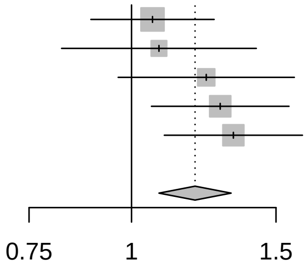

Heterogeneity: $I^2 = 3\%$, $\tau^2 = 0.0020$, $p = 0.39$

3.3.4 Baujat

```
baujat(sf_insitu_rma)
```

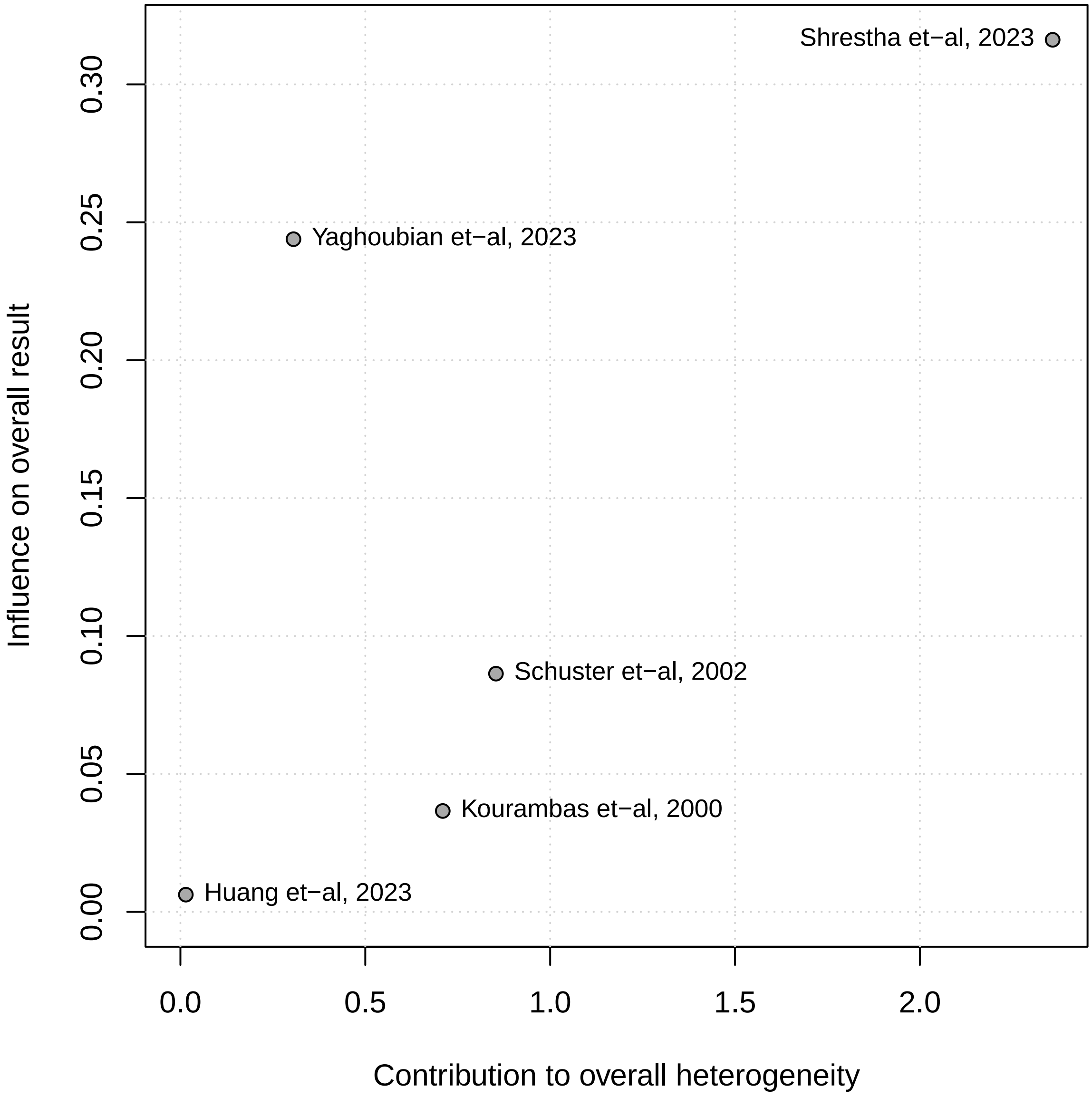

3.3.5 Leave One Out

```r
metainf(sf_insitu_rma)
```

```
## Influential analysis (common effect model)
##
##                                         RR           95%-CI p-value   tau^2
## Omitting Kourambas et-al, 2000    1.2272 [1.1039; 1.3643]  0.0002  0.0032
## Omitting Schuster et-al, 2002     1.1943 [1.0670; 1.3367]  0.0020  0.0006
## Omitting Yaghoubian et-al, 2023   1.1804 [1.0537; 1.3222]  0.0042  0.0035
## Omitting Shrestha et-al, 2023     1.2560 [1.1174; 1.4118]  0.0001  0.0000
## Omitting Huang et-al, 2023        1.2094 [1.0864; 1.3462]  0.0005  0.0044
##
## Pooled estimate                   1.2146 [1.0993; 1.3420]  0.0001  0.0020
##                                      tau    I^2
## Omitting Kourambas et-al, 2000     0.0561  15.0%
## Omitting Schuster et-al, 2002      0.0236   0.0%
## Omitting Yaghoubian et-al, 2023    0.0592  12.7%
## Omitting Shrestha et-al, 2023      0.0000   0.0%
## Omitting Huang et-al, 2023         0.0665  25.8%
##
## Pooled estimate                    0.0445   2.8%
##
## Details on meta-analytical method:
## - Mantel-Haenszel method
## - Restricted maximum-likelihood estimator for tau^2
```

3.4 RCT only Meta-Analysis (in situ (reference) vs displacement)

3.4.1 Result

```
rct_data <- insitu_data %>% subset(study_type == "Prospective Randomised Study ")
sf_insitu_rct_rma <- metabin(data = rct_data,
                   event.c = in_situ_stone_free_n,
                   n.c = number_in_situ,
                   event.e = displacement_stone_free_n,
                   n.e = number_displaced,
                   studlab = paste(author, year, sep = ", ")
                   )
sf_insitu_rct_rma
```

```
## Number of studies: k = 3
## Number of observations: o = 296
## Number of events: e = 232
##
##                          RR           95%-CI    z p-value
## Common effect model  1.2071 [1.0693; 1.3626] 3.04  0.0023
## Random effects model 1.1747 [1.0312; 1.3381] 2.42  0.0154
##
## Quantifying heterogeneity:
##  tau^2 = 0.0029 [0.0000; 0.3888]; tau = 0.0536 [0.0000; 0.6236]
##  I^2 = 12.3% [0.0%; 90.9%]; H = 1.07 [1.00; 3.31]
##
## Test of heterogeneity:
##     Q d.f. p-value
##  2.28    2  0.3196
##
## Details on meta-analytical method:
## - Mantel-Haenszel method
## - Inverse variance method
## - Restricted maximum-likelihood estimator for tau^2
## - Q-Profile method for confidence interval of tau^2 and tau
```

3.4.2 Forest plot

```r
forest(sf_insitu_rct_rma,
       sortvar = TE)
```

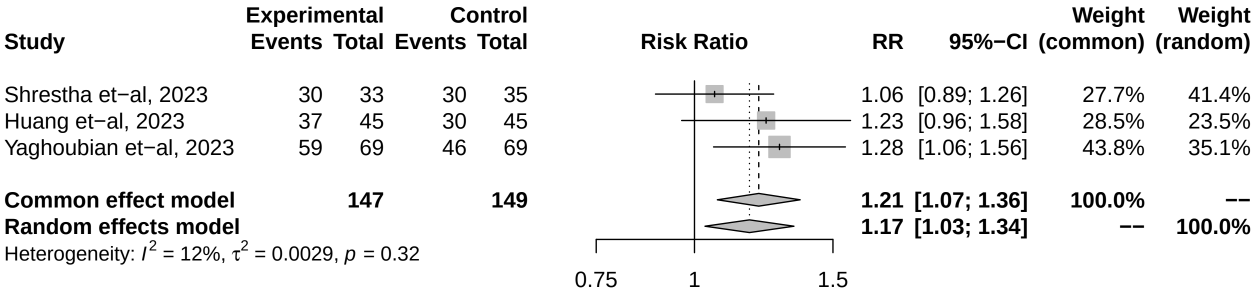

3.4.3 Trim and Fill

```r
trimfill(sf_insitu_rct_rma)
```

```
## Number of studies: k = 3 (with 0 added studies)
## Number of observations: o = 296
## Number of events: e = 232
##
##                          RR           95%-CI    z p-value
## Random effects model 1.1747 [1.0312; 1.3381] 2.42  0.0154
##
## Quantifying heterogeneity:
##  tau^2 = 0.0029 [0.0000; 0.3888]; tau = 0.0536 [0.0000; 0.6236]
##  I^2 = 12.3% [0.0%; 90.9%]; H = 1.07 [1.00; 3.31]
##
## Test of heterogeneity:
##     Q d.f. p-value
##  2.28    2  0.3196
##
## Details on meta-analytical method:
## - Inverse variance method
## - Restricted maximum-likelihood estimator for tau^2
## - Q-Profile method for confidence interval of tau^2 and tau
## - Trim-and-fill method to adjust for funnel plot asymmetry (L-estimator)
```

```r
funnel(trimfill(sf_insitu_rct_rma))
```

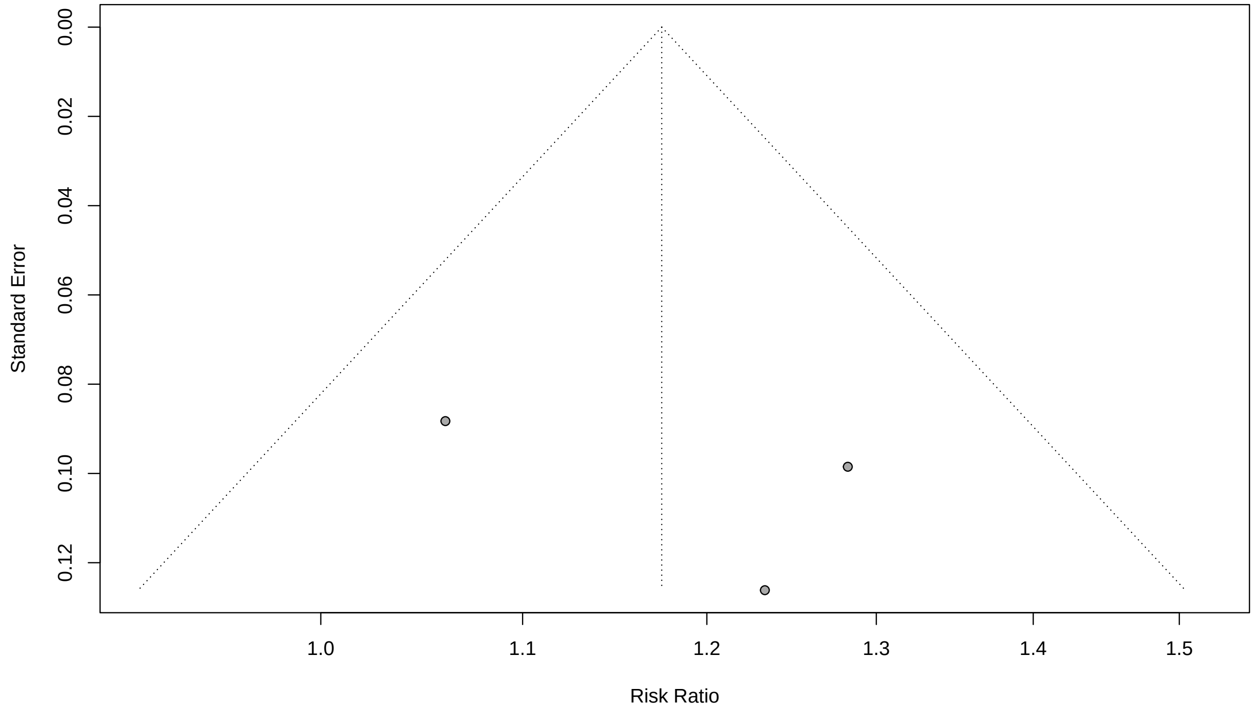


```r
forest(trimfill(sf_insitu_rct_rma), sortvar = TE)
```

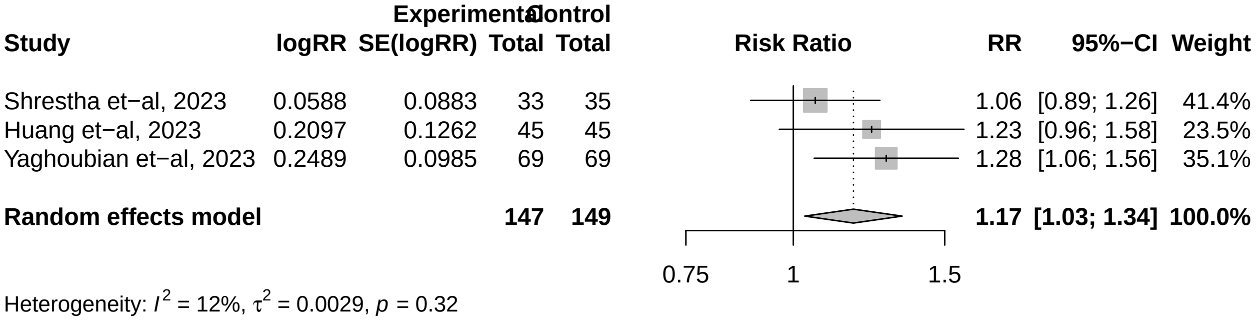
Experimental Control
Study logRR SE(logRR) Total Total
Risk Ratio
RR 95%−CI Weight
Shrestha et−al, 2023 0.0588 0.0883 33 35 1.06 [0.89; 1.26] 41.4%
Huang et−al, 2023 0.2097 0.1262 45 45 1.23 [0.96; 1.58] 23.5%
Yaghoubian et−al, 2023 0.2489 0.0985 69 69 1.28 [1.06; 1.56] 35.1%
Random effects model 147 149 1.17 [1.03; 1.34] 100.0%
0.75
1
1.5
Heterogeneity: I^2 = 12%, τ^2 = 0.0029, p = 0.32

3.4.4 Baujat

```r
baujat(sf_insitu_rct_rma)
```

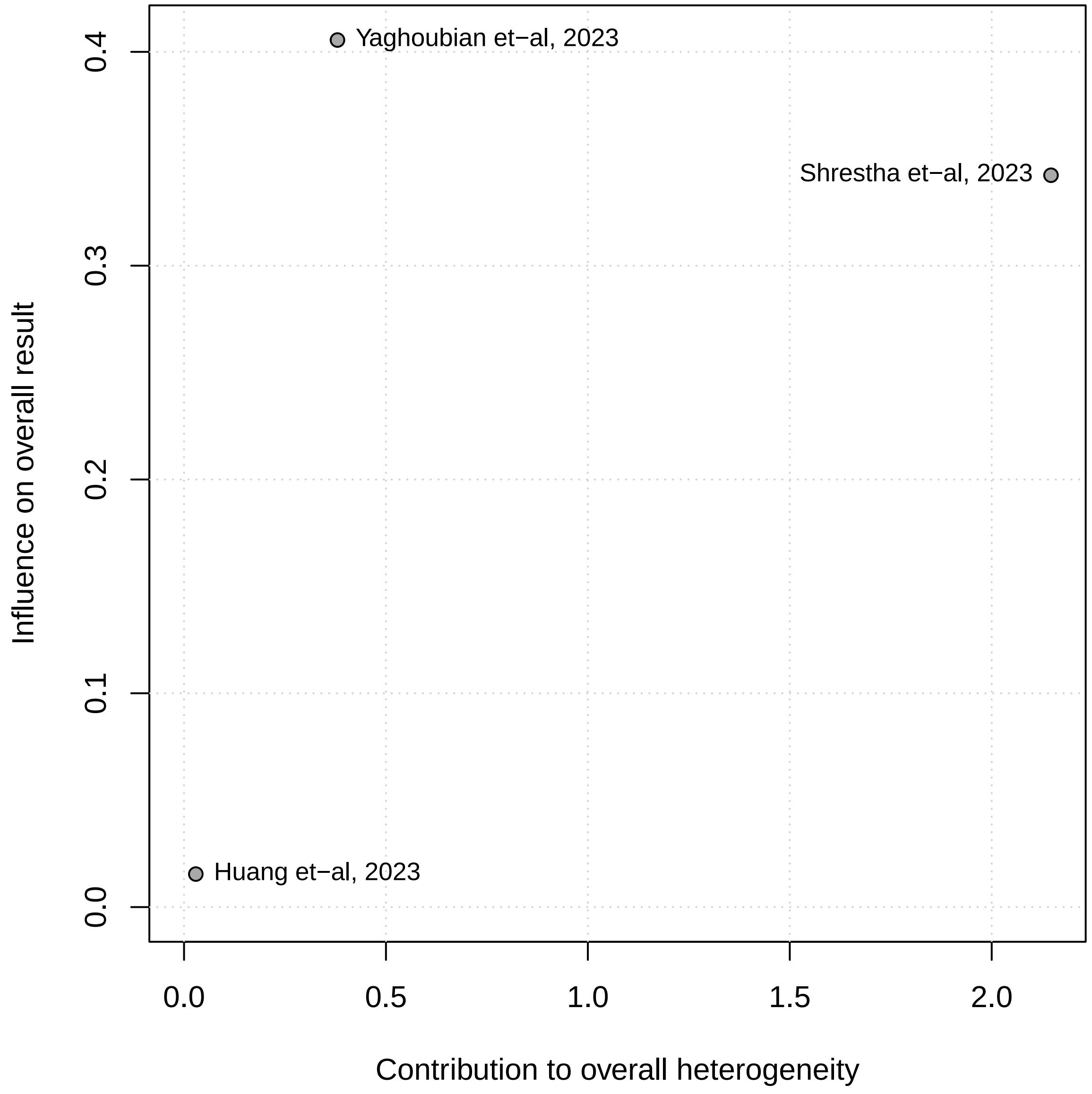

3.4.5 Leave One Out

```r
metainf(sf_insitu_rct_rma)
```

```
## Influential analysis (common effect model)
##
##                                       RR           95%-CI p-value   tau^2
## Omitting Yaghoubian et-al, 2023   1.1483 [0.9847; 1.3390]  0.0779  0.0000
## Omitting Shrestha et-al, 2023     1.2632 [1.0848; 1.4708]  0.0026  0.0000
## Omitting Huang et-al, 2023        1.1966 [1.0426; 1.3732]  0.0107  0.0093
##
## Pooled estimate                   1.2071 [1.0693; 1.3626]  0.0023  0.0029
##                                      tau    I^2
## Omitting Yaghoubian et-al, 2023    0.0000   0.0%
## Omitting Shrestha et-al, 2023      0.0000   0.0%
## Omitting Huang et-al, 2023         0.0965  51.6%
##
## Pooled estimate                    0.0536  12.3%
##
## Details on meta-analytical method:
## - Mantel-Haenszel method
## - Restricted maximum-likelihood estimator for tau^2
```

3.5 Stone Free definition Meta-Analysis (in situ (reference) vs displacement)

3.5.1 Result

```
sf_definition_data <- insitu_data[2:4,]
sf_definition_data_rma <- metabin(data = sf_definition_data,
                   event.c = in_situ_stone_free_n,
                   n.c = number_in_situ,
                   event.e = displacement_stone_free_n,
                   n.e = number_displaced,
                   studlab = paste(author, year, sep = ", ")
                   )
sf_definition_data_rma
```

```
## Number of studies: k = 3
## Number of observations: o = 284
## Number of events: e = 225
##
##                          RR           95%-CI    z p-value
## Common effect model  1.2253 [1.0918; 1.3752] 3.45  0.0006
## Random effects model 1.2116 [1.0502; 1.3978] 2.63  0.0085
##
## Quantifying heterogeneity:
##  tau^2 = 0.0069 [0.0000; 0.5774]; tau = 0.0832 [0.0000; 0.7598]
##  I^2 = 42.9% [0.0%; 82.8%]; H = 1.32 [1.00; 2.41]
##
## Test of heterogeneity:
##     Q d.f. p-value
##  3.50    2  0.1737
##
## Details on meta-analytical method:
## - Mantel-Haenszel method
## - Inverse variance method
## - Restricted maximum-likelihood estimator for tau^2
## - Q-Profile method for confidence interval of tau^2 and tau
```

3.5.2 Forest plot

```r
forest(sf_definition_data_rma,
       sortvar = TE)
```

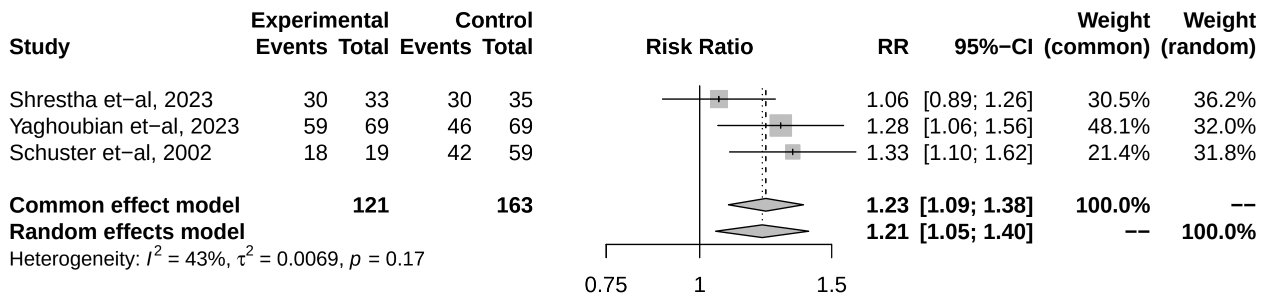

3.5.3 Trim and Fill

```r
trimfill(sf_definition_data_rma)
```

```
## Number of studies: k = 3 (with 0 added studies)
## Number of observations: o = 284
## Number of events: e = 225
##
##                            RR           95%-CI    z p-value
## Random effects model 1.2116 [1.0502; 1.3978] 2.63  0.0085
##
## Quantifying heterogeneity:
##  tau^2 = 0.0069 [0.0000; 0.5774]; tau = 0.0832 [0.0000; 0.7598]
##  I^2 = 42.9% [0.0%; 82.8%]; H = 1.32 [1.00; 2.41]
##
## Test of heterogeneity:
##     Q d.f. p-value
##  3.50    2  0.1737
##
## Details on meta-analytical method:
## - Inverse variance method
## - Restricted maximum-likelihood estimator for tau^2
## - Q-Profile method for confidence interval of tau^2 and tau
## - Trim-and-fill method to adjust for funnel plot asymmetry (L-estimator)
```

```r
funnel(trimfill(sf_definition_data_rma))
```

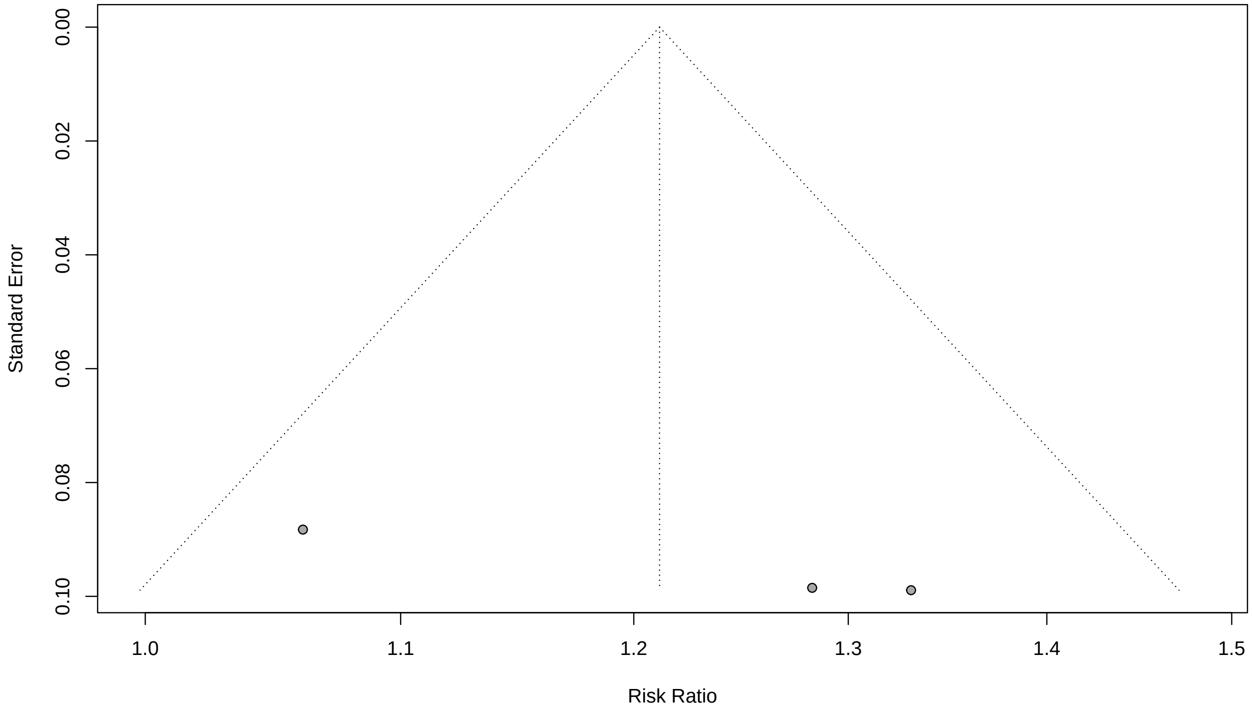


```r
forest(trimfill(sf_definition_data_rma), sortvar = TE)
```

Study	logRR	SE(logRR)	Experimental Total	Control Total	RR	95%−CI	Weight
Shrestha et−al, 2023	0.0588	0.0883	33	35	1.06	[0.89; 1.26]	36.2%
Yaghoubian et−al, 2023	0.2489	0.0985	69	69	1.28	[1.06; 1.56]	32.0%
Schuster et−al, 2002	0.2858	0.0989	19	59	1.33	[1.10; 1.62]	31.8%
**Random effects model**			**121**	**163**	**1.21**	**[1.05; 1.40]**	**100.0%**

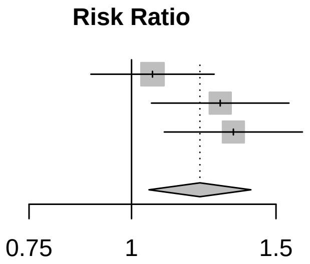

Heterogeneity: $I^2 = 43\%$, $\tau^2 = 0.0069$, $p = 0.17$

3.5.4 Baujat

```
baujat(sf_definition_data_rma)
```

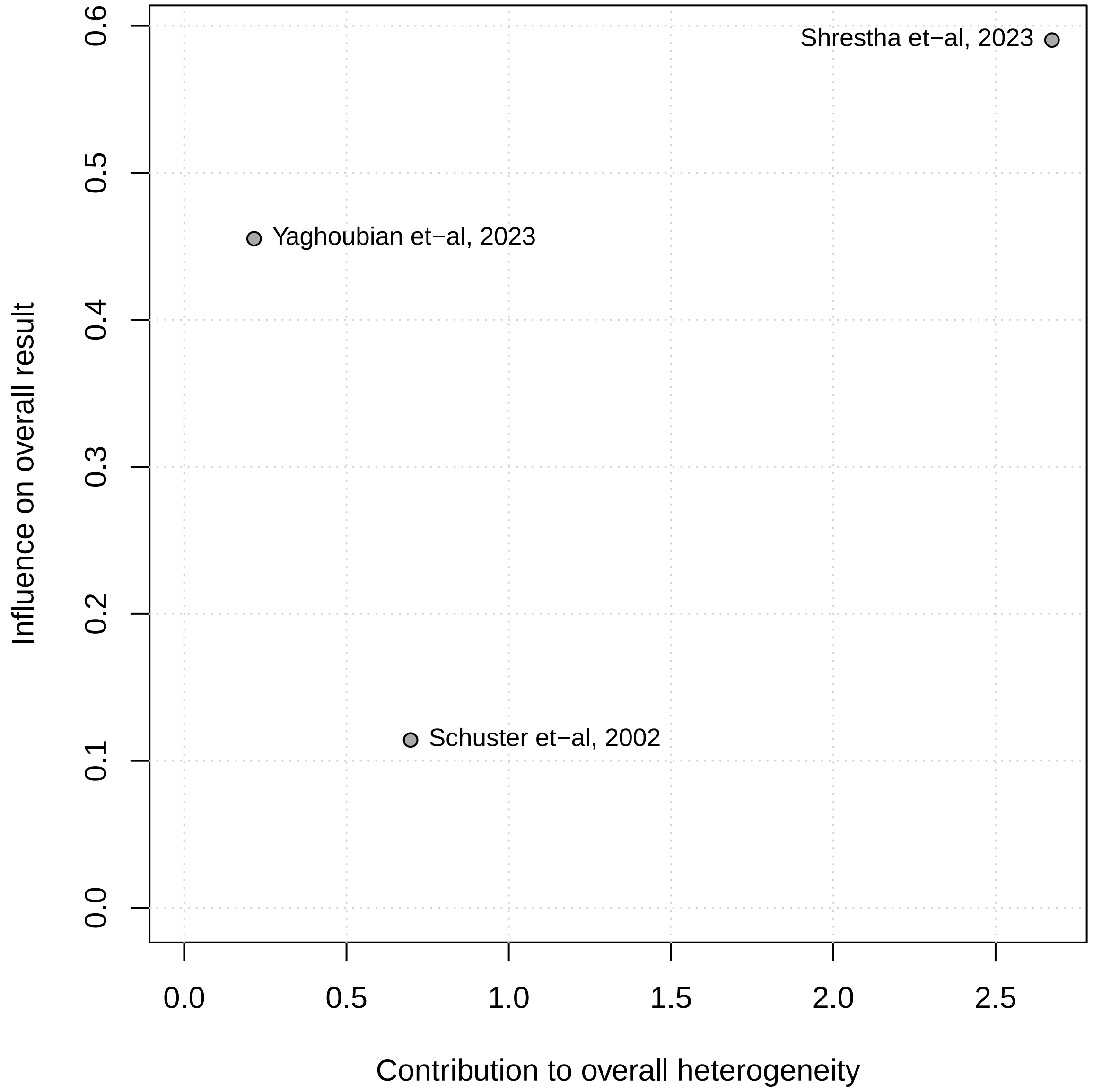

3.5.5 Leave One Out

```r
metainf(sf_definition_data_rma)
```

```
## Influential analysis (common effect model)
##
##                                         RR           95%-CI p-value   tau^2
## Omitting Schuster et-al, 2002     1.1966 [1.0426; 1.3732]  0.0107  0.0093
## Omitting Yaghoubian et-al, 2023   1.1721 [1.0304; 1.3334]  0.0157  0.0170
## Omitting Shrestha et-al, 2023     1.2975 [1.1212; 1.5014]  0.0005  0.0000
##
## Pooled estimate                   1.2253 [1.0918; 1.3752]  0.0006  0.0069
##                                      tau    I^2
## Omitting Schuster et-al, 2002      0.0965  51.6%
## Omitting Yaghoubian et-al, 2023    0.1303  65.9%
## Omitting Shrestha et-al, 2023      0.0000   0.0%
##
## Pooled estimate                    0.0832  42.9%
##
## Details on meta-analytical method:
## - Mantel-Haenszel method
## - Restricted maximum-likelihood estimator for tau^2
```

3.6 Stones 10mm-20mm Meta-Analysis (in situ (reference) vs displacement)

3.6.1 Result

```
stones_10_20_data <- insitu_data %>% subset(author != "Yaghoubian et-al")
stones_10_20_data_rma <- metabin(data = stones_10_20_data,
                   event.c = in_situ_stone_free_n,
                   n.c = number_in_situ,
                   event.e = displacement_stone_free_n,
                   n.e = number_displaced,
                   studlab = paste(author, year, sep = ", ")
                   )
stones_10_20_data_rma
```

```
## Number of studies: k = 4
## Number of observations: o = 270
## Number of events: e = 216
##
##                          RR           95%-CI    z p-value
## Common effect model  1.1804 [1.0537; 1.3222] 2.86  0.0042
## Random effects model 1.1710 [1.0367; 1.3228] 2.54  0.0111
##
## Quantifying heterogeneity:
##  tau^2 = 0.0035 [0.0000; 0.1527]; tau = 0.0592 [0.0000; 0.3908]
##  I^2 = 12.7% [0.0%; 86.6%]; H = 1.07 [1.00; 2.73]
##
## Test of heterogeneity:
##     Q d.f. p-value
##  3.44    3  0.3292
##
## Details on meta-analytical method:
## - Mantel-Haenszel method
## - Inverse variance method
## - Restricted maximum-likelihood estimator for tau^2
## - Q-Profile method for confidence interval of tau^2 and tau
```

3.6.2 Forest plot

```
forest(stones_10_20_data_rma,
       sortvar = TE)
```

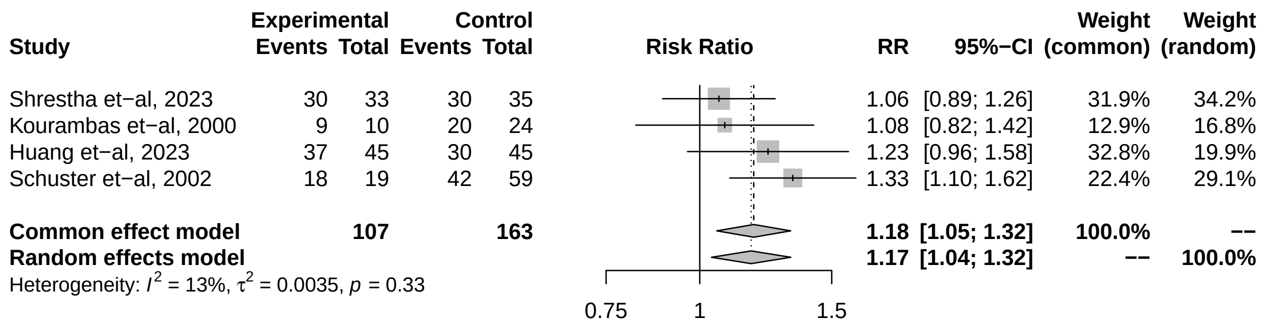

3.6.3 Trim and Fill

```r
trimfill(stones_10_20_data_rma)
```

```
## Number of studies: k = 4 (with 0 added studies)
## Number of observations: o = 270
## Number of events: e = 216
##
##                              RR           95%-CI    z p-value
## Random effects model 1.1710 [1.0367; 1.3228] 2.54  0.0111
##
## Quantifying heterogeneity:
##  tau^2 = 0.0035 [0.0000; 0.1527]; tau = 0.0592 [0.0000; 0.3908]
##  I^2 = 12.7% [0.0%; 86.6%]; H = 1.07 [1.00; 2.73]
##
## Test of heterogeneity:
##     Q d.f. p-value
##  3.44    3  0.3292
##
## Details on meta-analytical method:
## - Inverse variance method
## - Restricted maximum-likelihood estimator for tau^2
## - Q-Profile method for confidence interval of tau^2 and tau
## - Trim-and-fill method to adjust for funnel plot asymmetry (L-estimator)
```

```r
funnel(trimfill(stones_10_20_data_rma))
```

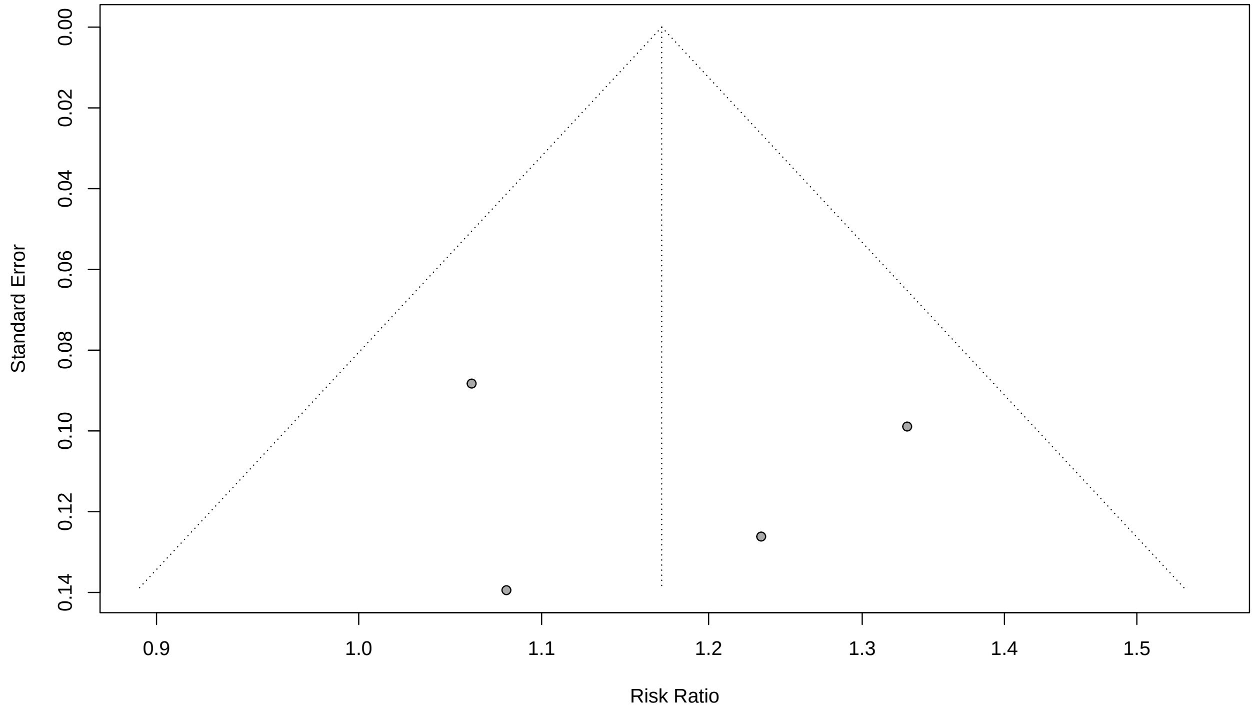


```r
forest(trimfill(stones_10_20_data_rma), sortvar = TE)
```

Study	logRR	SE(logRR)	Experimental Total	Control Total	RR	95%−CI	Weight
Shrestha et−al, 2023	0.0588	0.0883	33	35	1.06	[0.89; 1.26]	34.2%
Kourambas et−al, 2000	0.0770	0.1394	10	24	1.08	[0.82; 1.42]	16.8%
Huang et−al, 2023	0.2097	0.1262	45	45	1.23	[0.96; 1.58]	19.9%
Schuster et−al, 2002	0.2858	0.0989	19	59	1.33	[1.10; 1.62]	29.1%
**Random effects model**			**107**	**163**	**1.17**	**[1.04; 1.32]**	**100.0%**

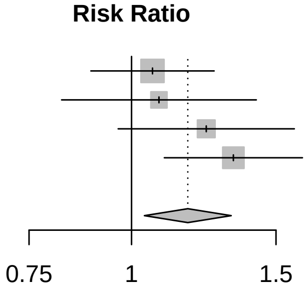

Heterogeneity: $I^2 = 13\%$, $\tau^2 = 0.0035$, $p = 0.33$

3.6.4 Baujat

```
baujat(stones_10_20_data_rma)
```

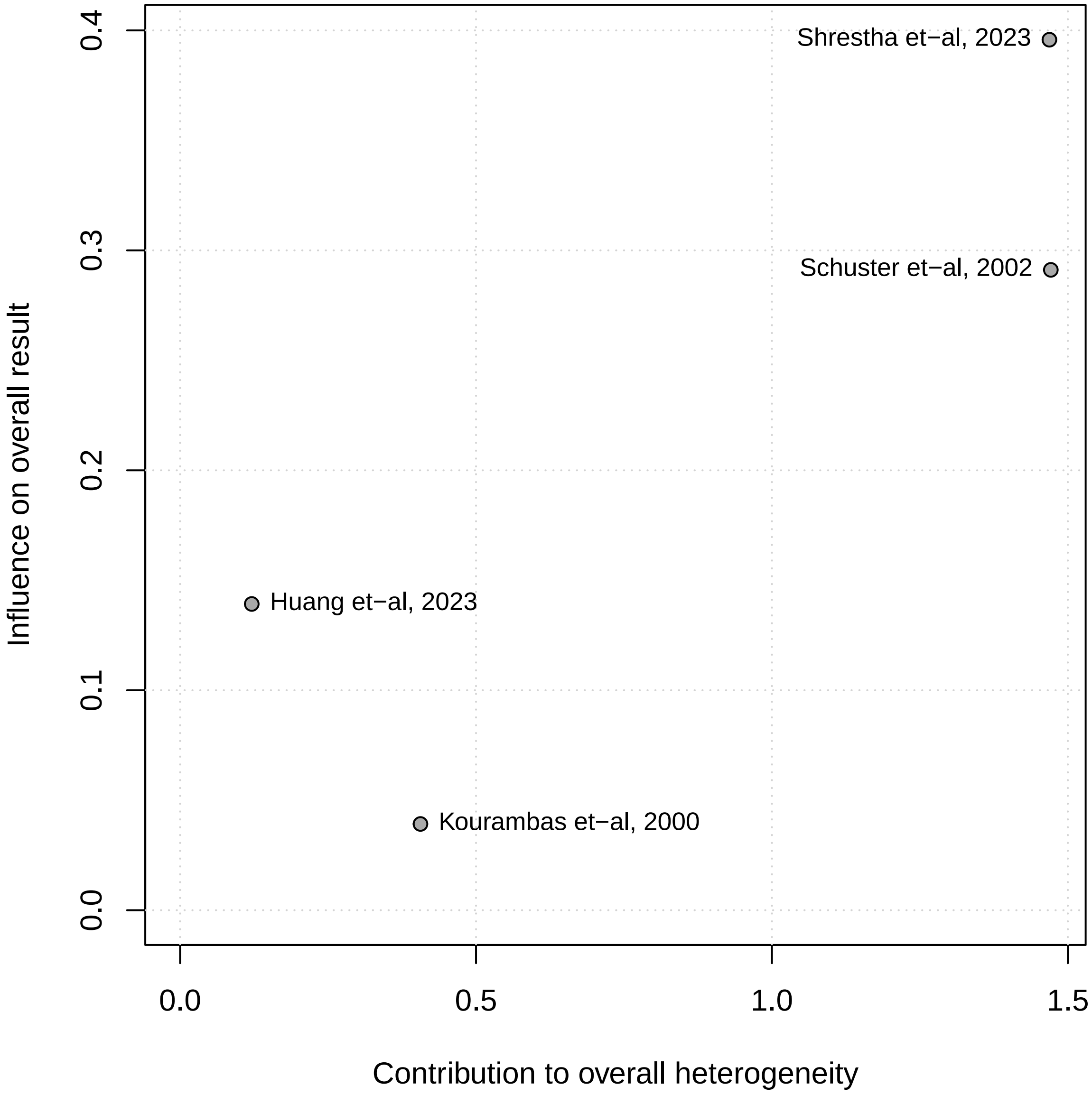

3.6.5 Leave One Out

```r
metainf(stones_10_20_data_rma)
```

```
## Influential analysis (common effect model)
##
##                                        RR           95%-CI p-value   tau^2
## Omitting Kourambas et-al, 2000   1.1952 [1.0562; 1.3525]  0.0047  0.0064
## Omitting Schuster et-al, 2002    1.1369 [0.9922; 1.3028]  0.0648  0.0000
## Omitting Shrestha et-al, 2023    1.2364 [1.0700; 1.4286]  0.0040  0.0000
## Omitting Huang et-al, 2023       1.1545 [1.0274; 1.2972]  0.0157  0.0075
##
## Pooled estimate                  1.1804 [1.0537; 1.3222]  0.0042  0.0035
##                                        tau    I^2
## Omitting Kourambas et-al, 2000    0.0803  34.5%
## Omitting Schuster et-al, 2002     0.0000   0.0%
## Omitting Shrestha et-al, 2023     0.0000   0.0%
## Omitting Huang et-al, 2023        0.0868  37.8%
##
## Pooled estimate                   0.0592  12.7%
##
## Details on meta-analytical method:
## - Mantel-Haenszel method
## - Restricted maximum-likelihood estimator for tau^2
```

4 Need for Stenting

4.1 Meta-analysis of Proportions for In Situ

```
in_situ_stent_metaprop <- meta::metaprop(
  data = insitu_data,
  event = in_situ_stenting_required_n,
  n = number_in_situ,
  studlab = paste(author, year, sep = ", "),
  method = "Inverse"
)
in_situ_stent_metaprop
```

```
## Number of studies: k = 5
## Number of observations: o = 232
## Number of events: e = 146
##
##                        proportion           95%-CI
## Common effect model        0.5977 [0.5147; 0.6755]
## Random effects model       0.7588 [0.2426; 0.9687]
##
## Quantifying heterogeneity:
##  tau^2 = 6.0184 [1.6101; 59.8708]; tau = 2.4532 [1.2689; 7.7376]
##  I^2 = 90.4% [80.6%; 95.3%]; H = 3.23 [2.27; 4.60]
##
## Test of heterogeneity:
##      Q d.f.  p-value
##  41.76    4 < 0.0001
##
## Details on meta-analytical method:
## - Inverse variance method
## - Restricted maximum-likelihood estimator for tau^2
## - Q-Profile method for confidence interval of tau^2 and tau
## - Logit transformation
## - Continuity correction of 0.5 in studies with zero cell frequencies
```

4.2 Meta-analysis of Proportions for Displacement

```
displacement_stent_metaprop <- metaprop(
  event = displacement_stenting_required_n,
  n = number_displaced,
  data = insitu_data,
  studlab = paste(author, year, sep = ", "),
  method = "Inverse"
)
displacement_stent_metaprop
```

```
## Number of studies: k = 5
## Number of observations: o = 176
## Number of events: e = 104
##
##                        proportion           95%-CI
## Common effect model        0.5494 [0.4510; 0.6440]
## Random effects model       0.6999 [0.2205; 0.9506]
##
## Quantifying heterogeneity:
##  tau^2 = 5.0053 [1.2529; 51.7163]; tau = 2.2372 [1.1193; 7.1914]
##  I^2 = 90.4% [80.6%; 95.3%]; H = 3.23 [2.27; 4.61]
##
## Test of heterogeneity:
##      Q d.f.  p-value
##  41.84    4 < 0.0001
##
## Details on meta-analytical method:
## - Inverse variance method
## - Restricted maximum-likelihood estimator for tau^2
## - Q-Profile method for confidence interval of tau^2 and tau
## - Logit transformation
## - Continuity correction of 0.5 in studies with zero cell frequencies
```

4.3 Meta-Analysis

4.3.1 Result

```r
stent_insitu_rma <- metabin(data = insitu_data,
                    event.c = in_situ_stenting_required_n,
                    n.c = number_in_situ,
                    event.e = displacement_stenting_required_n,
                    n.e = number_displaced,
                    studlab = paste(author, year, sep = ", ")
                    )
stent_insitu_rma
```

```
## Number of studies: k = 5
## Number of observations: o = 408
## Number of events: e = 250
##
##                          RR           95%-CI     z p-value
## Common effect model  0.9758 [0.8567; 1.1115] -0.37  0.7126
## Random effects model 1.0000 [0.9495; 1.0531] -0.00  0.9987
##
## Quantifying heterogeneity:
##  tau^2 = 0 [0.0000; 0.3401]; tau = 0 [0.0000; 0.5832]
##  I^2 = 0.0% [0.0%; 79.2%]; H = 1.00 [1.00; 2.19]
##
## Test of heterogeneity:
##     Q d.f. p-value
##  2.63    4  0.6217
##
## Details on meta-analytical method:
## - Mantel-Haenszel method
## - Inverse variance method
## - Restricted maximum-likelihood estimator for tau^2
## - Q-Profile method for confidence interval of tau^2 and tau
## - Continuity correction of 0.5 in studies with zero cell frequencies
```

4.3.2 Forest plot

```r
forest(stent_insitu_rma,
       sortvar = TE)
```

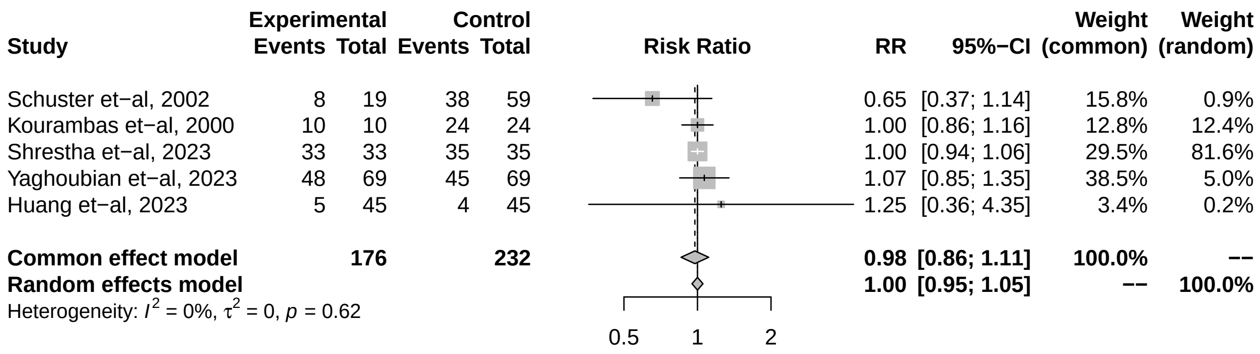

4.3.3 Trim and Fill

```r
trimfill(stent_insitu_rma)
```

```
## Number of studies: k = 5 (with 0 added studies)
## Number of observations: o = 408
## Number of events: e = 250
##
##                           RR           95%-CI     z p-value
## Random effects model 1.0000 [0.9495; 1.0531] -0.00  0.9987
##
## Quantifying heterogeneity:
##  tau^2 = 0 [0.0000; 0.3401]; tau = 0 [0.0000; 0.5832]
##  I^2 = 0.0% [0.0%; 79.2%]; H = 1.00 [1.00; 2.19]
##
## Test of heterogeneity:
##     Q d.f. p-value
##  2.63    4  0.6217
##
## Details on meta-analytical method:
## - Inverse variance method
## - Restricted maximum-likelihood estimator for tau^2
## - Q-Profile method for confidence interval of tau^2 and tau
## - Trim-and-fill method to adjust for funnel plot asymmetry (L-estimator)
```

```r
funnel(trimfill(stent_insitu_rma))
```

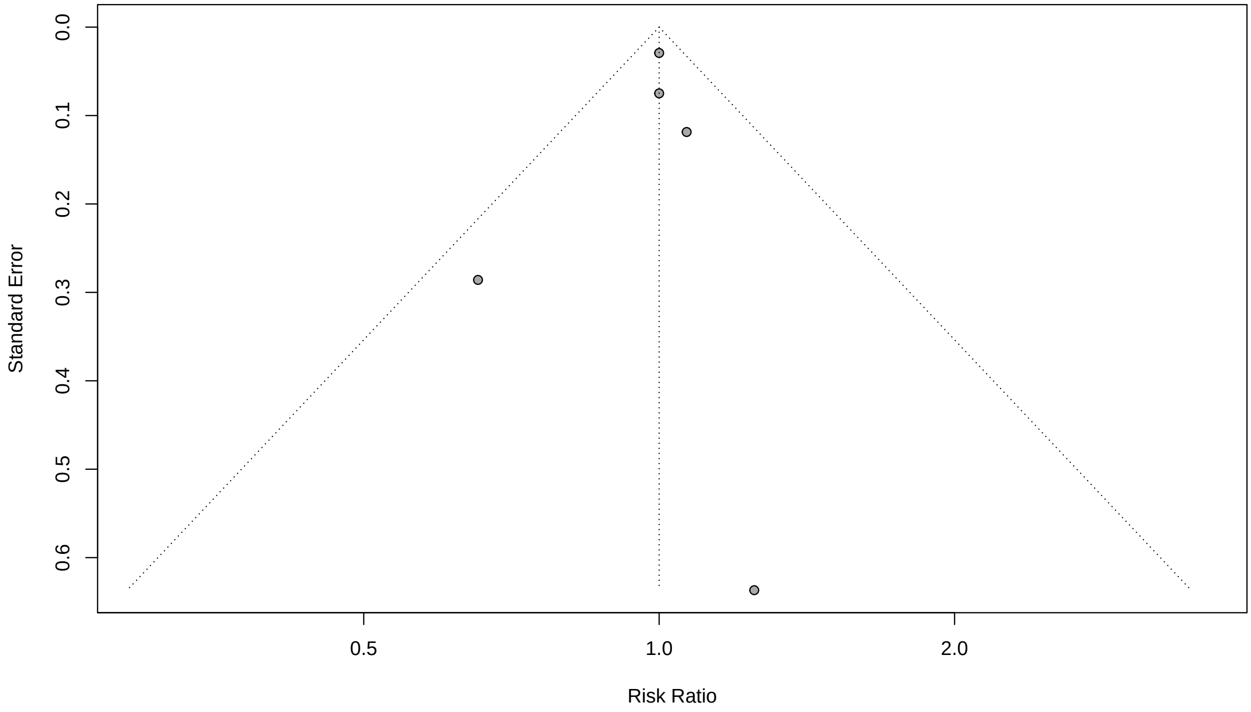


```r
forest(trimfill(stent_insitu_rma), sortvar = TE)
```

Study	logRR	SE(logRR)	Experimental Total	Control Total	RR	95%−CI	Weight
Schuster et−al, 2002	−0.4250	0.2859	19	59	0.65	[0.37; 1.14]	0.9%
Kourambas et−al, 2000	0.0000	0.0749	10	24	1.00	[0.86; 1.16]	12.4%
Shrestha et−al, 2023	0.0000	0.0292	33	35	1.00	[0.94; 1.06]	81.6%
Yaghoubian et−al, 2023	0.0645	0.1186	69	69	1.07	[0.85; 1.35]	5.0%
Huang et−al, 2023	0.2231	0.6368	45	45	1.25	[0.36; 4.35]	0.2%
**Random effects model**			**176**	**232**	**1.00**	**[0.95; 1.05]**	**100.0%**

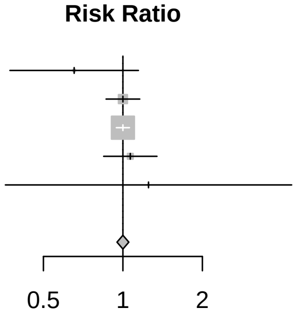

Heterogeneity: $I^2 = 0\%$, $\tau^2 = 0$, $p = 0.62$

4.3.4 Baujat

```
baujat(stent_insitu_rma)
```

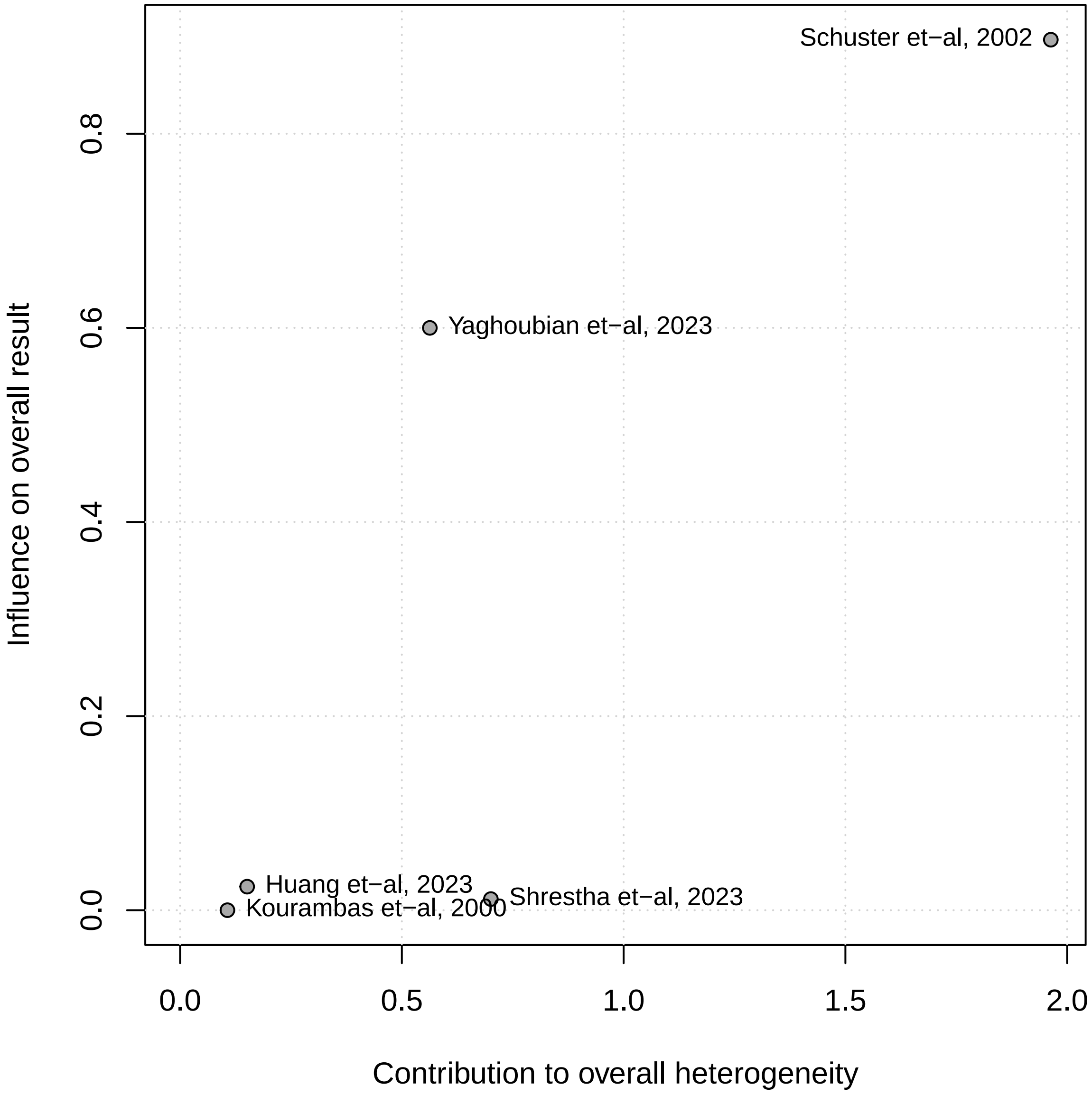

4.3.5 Leave One Out

```r
metainf(stent_insitu_rma)
```

```
## Influential analysis (common effect model)
##
##                                       RR           95%-CI p-value   tau^2
## Omitting Kourambas et-al, 2000    0.9761 [0.8419; 1.1316]  0.7484  0.0000
## Omitting Schuster et-al, 2002     1.0364 [0.9150; 1.1739]  0.5740  0.0000
## Omitting Yaghoubian et-al, 2023   0.9190 [0.7896; 1.0696]  0.2754  0.0000
## Omitting Shrestha et-al, 2023     0.9661 [0.8037; 1.1612]  0.7131  0.0000
## Omitting Huang et-al, 2023        0.9661 [0.8519; 1.0957]  0.5913  0.0000
##
## Pooled estimate                   0.9758 [0.8567; 1.1115]  0.7126  0.0000
##                                      tau   I^2
## Omitting Kourambas et-al, 2000     0.0000  0.0%
## Omitting Schuster et-al, 2002      0.0000  0.0%
## Omitting Yaghoubian et-al, 2023    0.0000  0.0%
## Omitting Shrestha et-al, 2023      0.0000  0.0%
## Omitting Huang et-al, 2023         0.0018  0.0%
##
## Pooled estimate                    0.0000  0.0%
##
## Details on meta-analytical method:
## - Mantel-Haenszel method
## - Restricted maximum-likelihood estimator for tau^2
```

5 Complications

5.1 Overall

5.1.1 Data Exploration

```
insitu_data_com <- insitu_data %>% drop_na(in_situ_complications_n)

insitu_data_com %>% subset(select = c(author,
                                      in_situ_complications_n,
                                      displaced_complications_n,
                                      in_situ_clavien_dindo_1,
                                      displaced_clavien_dindo_1,
                                      in_situ_clavien_dindo_2,
                                      displaced_clavien_dindo_2,
                                      in_situ_clavien_dindo_3,
                                      displaced_clavien_dindo_3,
                                      in_situ_clavien_dindo_4,
                                      displaced_clavien_dindo_4)) %>% gt()
```

author	in_situ_complications_n	displaced_complications_n	in_situ_clavien_dindo_1	displaced_clavi
Kourambas et-al	1	0	0	[illegible]
Schuster et-al	7	4	NA	[illegible]
Yaghoubian et-al	4	8	0	[illegible]
Shrestha et-al	5	6	4	[illegible]
Huang et-al	4	5	3	[illegible]

5.1.2 Meta-analysis of Proportions for In Situ

```r
in_situ_comp_metaprop <- meta::metaprop(
  data = insitu_data_com,
  event = in_situ_complications_n,
  n = number_in_situ,
  studlab = paste(author, year, sep = ", "),
  method = "Inverse"
)
in_situ_comp_metaprop
```

```
## Number of studies: k = 5
## Number of observations: o = 232
## Number of events: e = 21
##
##                        proportion           95%-CI
## Common effect model        0.0968 [0.0639; 0.1441]
## Random effects model       0.0968 [0.0639; 0.1441]
##
## Quantifying heterogeneity:
##  tau^2 = 0 [0.0000; 1.9858]; tau = 0 [0.0000; 1.4092]
##  I^2 = 0.0% [0.0%; 79.2%]; H = 1.00 [1.00; 2.19]
##
## Test of heterogeneity:
##     Q d.f.  p-value
##  3.13    4  0.5366
##
## Details on meta-analytical method:
## - Inverse variance method
## - Restricted maximum-likelihood estimator for tau^2
## - Q-Profile method for confidence interval of tau^2 and tau
## - Logit transformation
```

5.1.3 Meta-analysis of Proportions for Displacement

```
displacement_comp_metaprop <- metaprop(
  event = displaced_complications_n,
  n = number_displaced,
  data = insitu_data_com,
  studlab = paste(author, year, sep = ", "),
  method = "Inverse"
)
displacement_comp_metaprop
```

```
## Number of studies: k = 5
## Number of observations: o = 176
## Number of events: e = 23
##
##                       proportion           95%-CI
## Common effect model       0.1388 [0.0942; 0.1998]
## Random effects model      0.1388 [0.0942; 0.1998]
##
## Quantifying heterogeneity:
##  tau^2 = 0 [0.0000; 2.4672]; tau = 0 [0.0000; 1.5707]
##  I^2 = 0.0% [0.0%; 79.2%]; H = 1.00 [1.00; 2.19]
##
## Test of heterogeneity:
##     Q d.f. p-value
##  2.60    4  0.6261
##
## Details on meta-analytical method:
## - Inverse variance method
## - Restricted maximum-likelihood estimator for tau^2
## - Q-Profile method for confidence interval of tau^2 and tau
## - Logit transformation
## - Continuity correction of 0.5 in studies with zero cell frequencies
```

5.1.4 Overall Meta-Analysis

```
comp_insitu_rma <- metabin(data = insitu_data_com,
                   event.c = in_situ_complications_n,
                   n.c = number_in_situ,
                   event.e = displaced_complications_n,
                   n.e = number_displaced,
                   studlab = paste(author, year, sep = ", ")
                   )
comp_insitu_rma
```

```
## Number of studies: k = 5
## Number of observations: o = 408
## Number of events: e = 44
##
##                          RR           95%-CI    z p-value
## Common effect model  1.5089 [0.8596; 2.6484] 1.43  0.1518
## Random effects model 1.5135 [0.8614; 2.6592] 1.44  0.1495
##
## Quantifying heterogeneity:
##  tau^2 = 0 [0.0000; 0.1804]; tau = 0 [0.0000; 0.4247]
##  I^2 = 0.0% [0.0%; 79.2%]; H = 1.00 [1.00; 2.19]
##
## Test of heterogeneity:
##     Q d.f. p-value
##  0.67    4  0.9555
##
## Details on meta-analytical method:
## - Mantel-Haenszel method
## - Inverse variance method
## - Restricted maximum-likelihood estimator for tau^2
## - Q-Profile method for confidence interval of tau^2 and tau
## - Continuity correction of 0.5 in studies with zero cell frequencies
```

5.1.5 Forest plot

```r
forest(comp_insitu_rma,
       sortvar = TE)
```

Study	Experimental Events	Experimental Total	Control Events	Control Total	Risk Ratio	RR	95%−CI	Weight (common)	Weight (random)
Kourambas et−al, 2000	0	10	1	24		0.78	[0.03; 17.58]	5.3%	3.3%
Huang et−al, 2023	5	45	4	45		1.25	[0.36; 4.35]	23.3%	20.4%
Shrestha et−al, 2023	6	33	5	35		1.27	[0.43; 3.78]	28.2%	26.9%
Schuster et−al, 2002	4	19	7	59		1.77	[0.58; 5.41]	19.9%	25.6%
Yaghoubian et−al, 2023	8	69	4	69		2.00	[0.63; 6.33]	23.3%	23.9%
**Common effect model**		**176**		**232**		**1.51**	**[0.86; 2.65]**	**100.0%**	**−−**
**Random effects model**						**1.51**	**[0.86; 2.66]**	**−−**	**100.0%**

Heterogeneity: $I^2 = 0\%$, $\tau^2 = 0$, $p = 0.96$

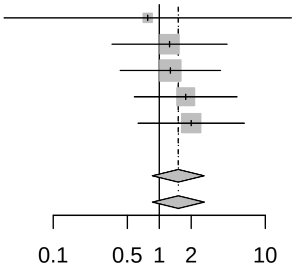

5.1.6 Trim and Fill

```
trimfill(comp_insitu_rma)
```

```
## Number of studies: k = 6 (with 1 added studies)
## Number of observations: o = 442
## Number of events: e = 45
##
##                           RR           95%-CI    z p-value
## Random effects model 1.5479 [0.8889; 2.6953] 1.54  0.1226
##
## Quantifying heterogeneity:
##  tau^2 = 0 [0.0000; 0.0187]; tau = 0 [0.0000; 0.1368]
##  I^2 = 0.0% [0.0%; 74.6%]; H = 1.00 [1.00; 1.99]
##
## Test of heterogeneity:
##     Q d.f. p-value
##  0.86    5  0.9731
##
## Details on meta-analytical method:
## - Inverse variance method
## - Restricted maximum-likelihood estimator for tau^2
## - Q-Profile method for confidence interval of tau^2 and tau
## - Trim-and-fill method to adjust for funnel plot asymmetry (L-estimator)
```

```
funnel(trimfill(comp_insitu_rma))
```

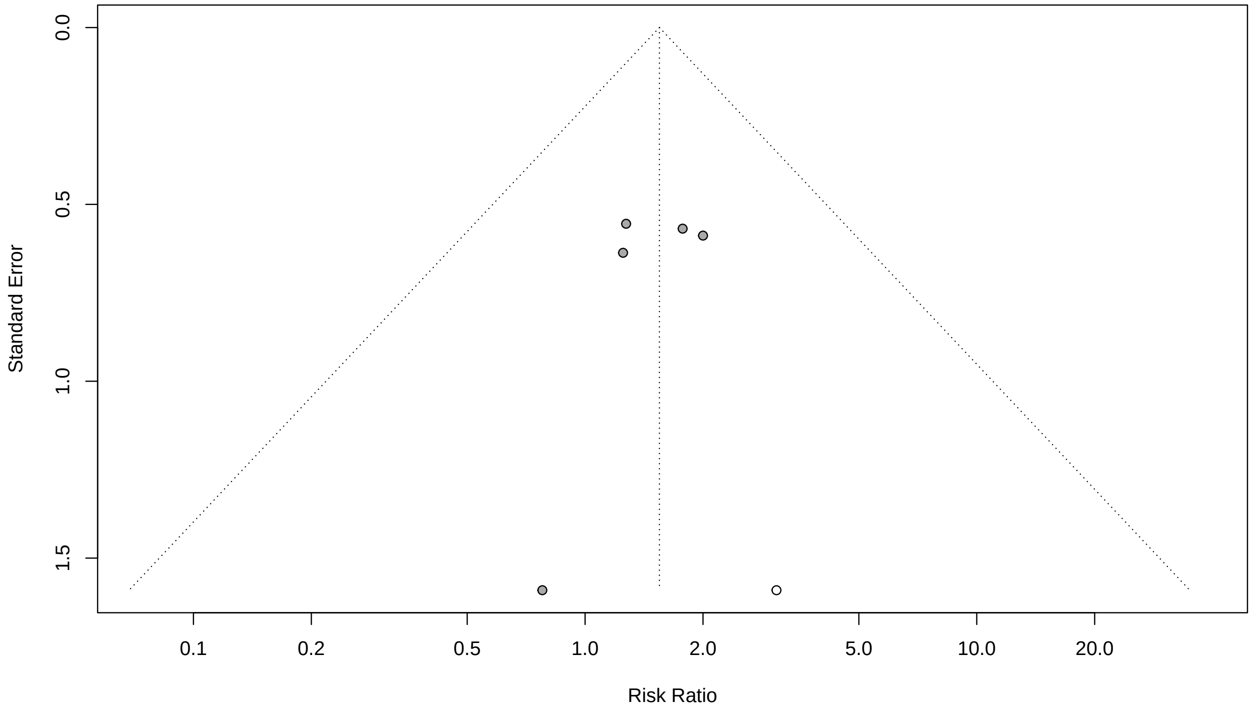


```
forest(trimfill(comp_insitu_rma), sortvar = TE)
```

Study	logRR	SE(logRR)	Experimental Total	Control Total	RR	95%−CI	Weight
Kourambas et−al, 2000	−0.2513	1.5908	10	24	0.78	[0.03; 17.58]	3.2%
Huang et−al, 2023	0.2231	0.6368	45	45	1.25	[0.36; 4.35]	19.7%
Shrestha et−al, 2023	0.2412	0.5548	33	35	1.27	[0.43; 3.78]	26.0%
Schuster et−al, 2002	0.5735	0.5686	19	59	1.77	[0.58; 5.41]	24.8%
Yaghoubian et−al, 2023	0.6931	0.5882	69	69	2.00	[0.63; 6.33]	23.1%
Filled: Kourambas et−al, 2000	1.1251	1.5908	10	24	3.08	[0.14; 69.62]	3.2%
**Random effects model**			**186**	**256**	**1.55**	**[0.89; 2.70]**	**100.0%**

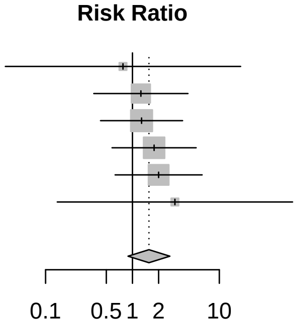

Heterogeneity: $I^2 = 0\%$, $\tau^2 = 0$, $p = 0.97$

5.1.7 Baujat

```
baujat(comp_insitu_rma)
```

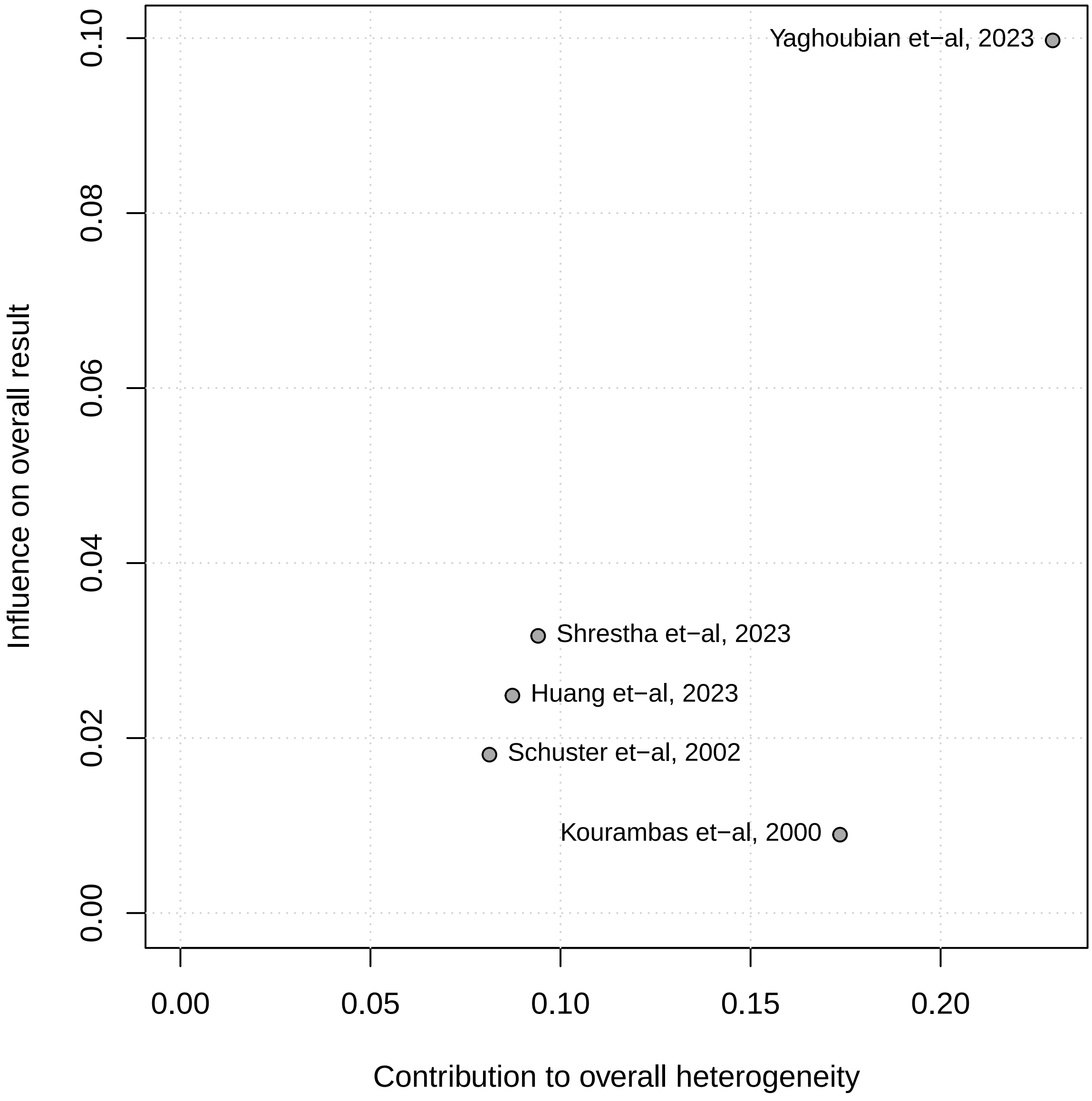

5.1.8 Leave One Out

```r
metainf(comp_insitu_rma)
```

```
## Influential analysis (common effect model)
##
##                                     RR           95%-CI p-value   tau^2
## Omitting Kourambas et-al, 2000    1.5512 [0.8745; 2.7515]  0.1332  0.0000
## Omitting Schuster et-al, 2002     1.4431 [0.7539; 2.7622]  0.2682  0.0000
## Omitting Yaghoubian et-al, 2023   1.3598 [0.7131; 2.5931]  0.3507  0.0000
## Omitting Shrestha et-al, 2023     1.6018 [0.8292; 3.0943]  0.1608  0.0000
## Omitting Huang et-al, 2023        1.5874 [0.8446; 2.9837]  0.1512  0.0000
##
## Pooled estimate                   1.5089 [0.8596; 2.6484]  0.1518  0.0000
##                                      tau   I^2
## Omitting Kourambas et-al, 2000     0.0000  0.0%
## Omitting Schuster et-al, 2002      0.0000  0.0%
## Omitting Yaghoubian et-al, 2023    0.0000  0.0%
## Omitting Shrestha et-al, 2023      0.0000  0.0%
## Omitting Huang et-al, 2023         0.0000  0.0%
##
## Pooled estimate                    0.0000  0.0%
##
## Details on meta-analytical method:
## - Mantel-Haenszel method
## - Restricted maximum-likelihood estimator for tau^2
```

5.2 Clavien Dindo I-II

5.2.1 Meta-analysis of Proportions for In Situ

```
insitu_data_com <- insitu_data_com %>% mutate(in_situ_cd_I_II = in_situ_clavien_dindo_1 + in_situ_clavie
                                              displacement_cd_I_II = displaced_clavien_dindo_1 + displac
                                              in_situ_cd_III = in_situ_clavien_dindo_3 + in_situ_clavien
                                              displacement_cd_III = displaced_clavien_dindo_3 + displace

                                              )

insitu_data_com_I_II <- insitu_data_com %>% drop_na(in_situ_cd_I_II)

in_situ_comp_metaprop <- meta::metaprop(
  data = insitu_data_com_I_II,
  event = in_situ_cd_I_II,
  n = number_in_situ,
  studlab = paste(author, year, sep = ", "),
  method = "Inverse"
)
in_situ_comp_metaprop
```

```
## Number of studies: k = 4
## Number of observations: o = 173
## Number of events: e = 13
##
##                       proportion           95%-CI
## Common effect model       0.0844 [0.0495; 0.1403]
## Random effects model      0.0818 [0.0440; 0.1470]
##
## Quantifying heterogeneity:
##  tau^2 = 0.0972 [0.0000; 5.3716]; tau = 0.3118 [0.0000; 2.3177]
##  I^2 = 14.1% [0.0%; 86.8%]; H = 1.08 [1.00; 2.76]
##
## Test of heterogeneity:
##     Q d.f. p-value
##  3.49    3  0.3220
##
## Details on meta-analytical method:
## - Inverse variance method
## - Restricted maximum-likelihood estimator for tau^2
## - Q-Profile method for confidence interval of tau^2 and tau
## - Logit transformation
```

5.2.2 Meta-analysis of Proportions for Displacement

```
displacement_comp_metaprop <- metaprop(
  event = displacement_cd_I_II,
  n = number_displaced,
  data = insitu_data_com_I_II,
  studlab = paste(author, year, sep = ", "),
  method = "Inverse"
)
displacement_comp_metaprop
```

```
## Number of studies: k = 4
## Number of observations: o = 157
## Number of events: e = 15
##
##                        proportion           95%-CI
## Common effect model        0.1099 [0.0677; 0.1737]
## Random effects model       0.1039 [0.0548; 0.1883]
##
## Quantifying heterogeneity:
##  tau^2 = 0.1504 [0.0000; 7.2560]; tau = 0.3879 [0.0000; 2.6937]
##  I^2 = 25.0% [0.0%; 71.1%]; H = 1.15 [1.00; 1.86]
##
## Test of heterogeneity:
##     Q d.f. p-value
##  4.00    3  0.2614
##
## Details on meta-analytical method:
## - Inverse variance method
## - Restricted maximum-likelihood estimator for tau^2
## - Q-Profile method for confidence interval of tau^2 and tau
## - Logit transformation
## - Continuity correction of 0.5 in studies with zero cell frequencies
```

5.2.3 Meta-Analysis

```
comp_insitu_rma <- metabin(data = insitu_data_com_I_II,
                  event.c = in_situ_cd_I_II,
                  n.c = number_in_situ,
                  event.e = displacement_cd_I_II,
                  n.e = number_displaced,
                  studlab = paste(author, year, sep = ", ")
                  )
comp_insitu_rma
```

```
## Number of studies: k = 4
## Number of observations: o = 330
## Number of events: e = 28
##
##                           RR           95%-CI    z p-value
## Common effect model  1.2429 [0.6170; 2.5037] 0.61  0.5429
## Random effects model 1.2445 [0.6024; 2.5710] 0.59  0.5545
##
## Quantifying heterogeneity:
##  tau^2 = 0 [0.0000; 5.4298]; tau = 0 [0.0000; 2.3302]
##  I^2 = 0.0% [0.0%; 84.7%]; H = 1.00 [1.00; 2.56]
##
## Test of heterogeneity:
##     Q d.f. p-value
##  2.15    3  0.5417
##
## Details on meta-analytical method:
## - Mantel-Haenszel method
## - Inverse variance method
## - Restricted maximum-likelihood estimator for tau^2
## - Q-Profile method for confidence interval of tau^2 and tau
## - Continuity correction of 0.5 in studies with zero cell frequencies
```

5.2.4 Forest plot

```r
forest(comp_insitu_rma,
       sortvar = TE)
```

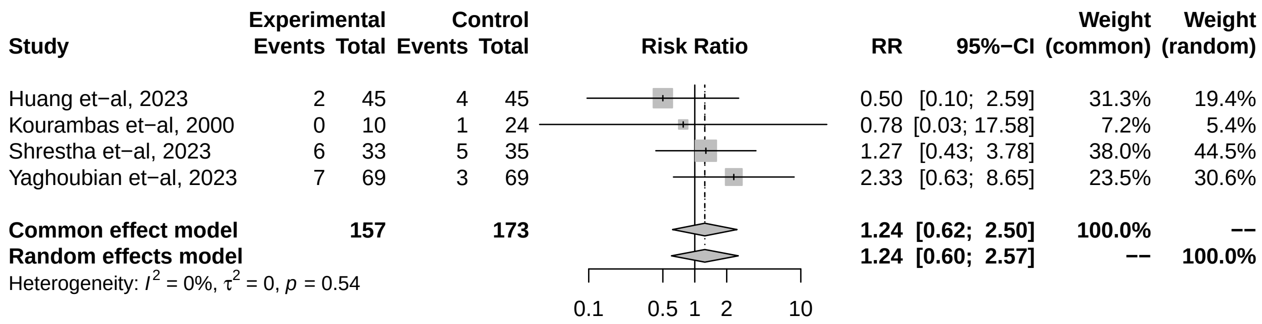

5.2.5 Trim and Fill

```r
trimfill(comp_insitu_rma)
```

```
## Number of studies: k = 6 (with 2 added studies)
## Number of observations: o = 454
## Number of events: e = 35
##
##                          RR           95%-CI    z p-value
## Random effects model 1.6316 [0.8432; 3.1573] 1.45  0.1461
##
## Quantifying heterogeneity:
##  tau^2 = 0.0154 [0.0000; 3.8338]; tau = 0.1242 [0.0000; 1.9580]
##  I^2 = 0.0% [0.0%; 74.6%]; H = 1.00 [1.00; 1.99]
##
## Test of heterogeneity:
##     Q d.f. p-value
##  4.88    5  0.4313
##
## Details on meta-analytical method:
## - Inverse variance method
## - Restricted maximum-likelihood estimator for tau^2
## - Q-Profile method for confidence interval of tau^2 and tau
## - Trim-and-fill method to adjust for funnel plot asymmetry (L-estimator)
```

```r
funnel(trimfill(comp_insitu_rma))
```

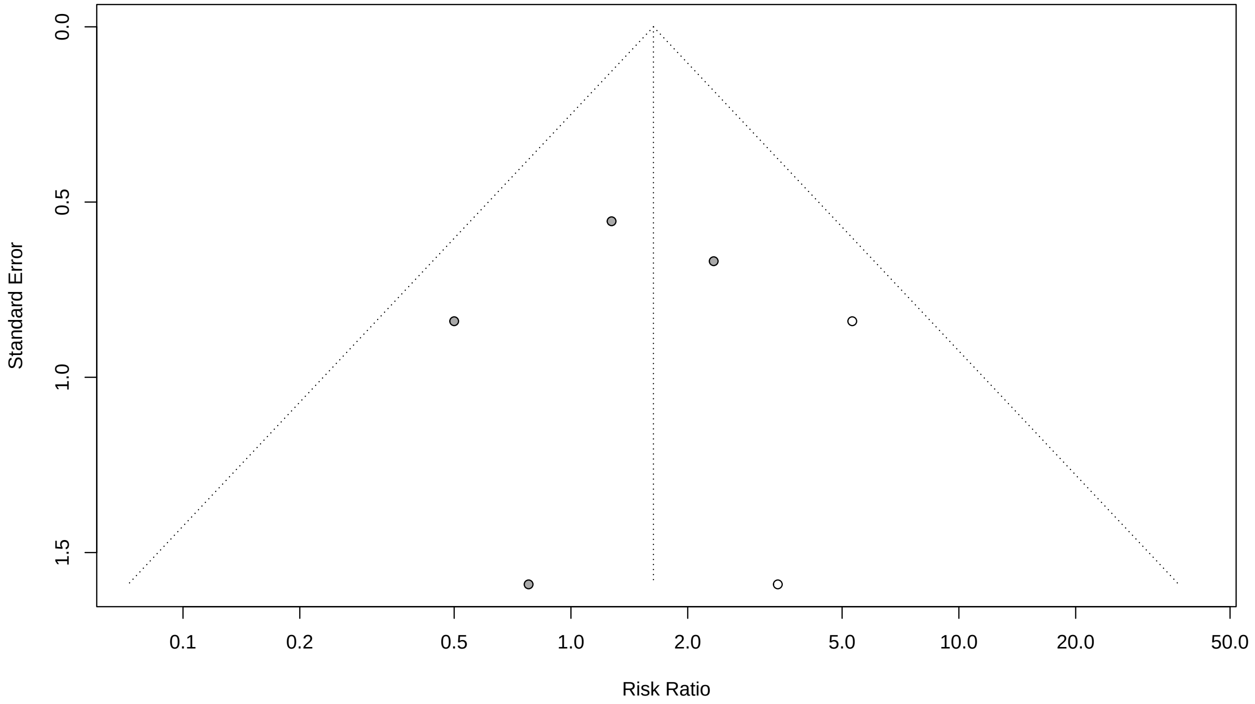


```r
forest(trimfill(comp_insitu_rma), sortvar = TE)
```

Study	logRR	SE(logRR)	Experimental Total	Control Total	Risk Ratio	RR	95%−CI	Weight
Huang et−al, 2023	−0.6931	0.8400	45	45		0.50	[0.10; 2.59]	15.7%
Kourambas et−al, 2000	−0.2513	1.5908	10	24		0.78	[0.03; 17.58]	4.5%
Shrestha et−al, 2023	0.2412	0.5548	33	35		1.27	[0.43; 3.78]	35.1%
Yaghoubian et−al, 2023	0.8473	0.6687	69	69		2.33	[0.63; 8.65]	24.5%
Filled: Kourambas et−al, 2000	1.2278	1.5908	10	24		3.41	[0.15; 77.15]	4.5%
Filled: Huang et−al, 2023	1.6697	0.8400	45	45		5.31	[1.02; 27.55]	15.7%
**Random effects model**			**212**	**242**		**1.63**	**[0.84; 3.16]**	**100.0%**

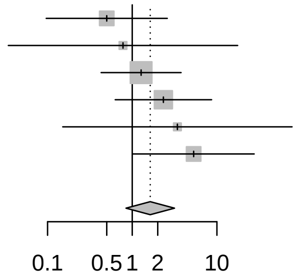

Heterogeneity: $I^2 = 0\%$, $\tau^2 = 0.0154$, $p = 0.43$

5.2.6 Baujat

```
baujat(comp_insitu_rma)
```

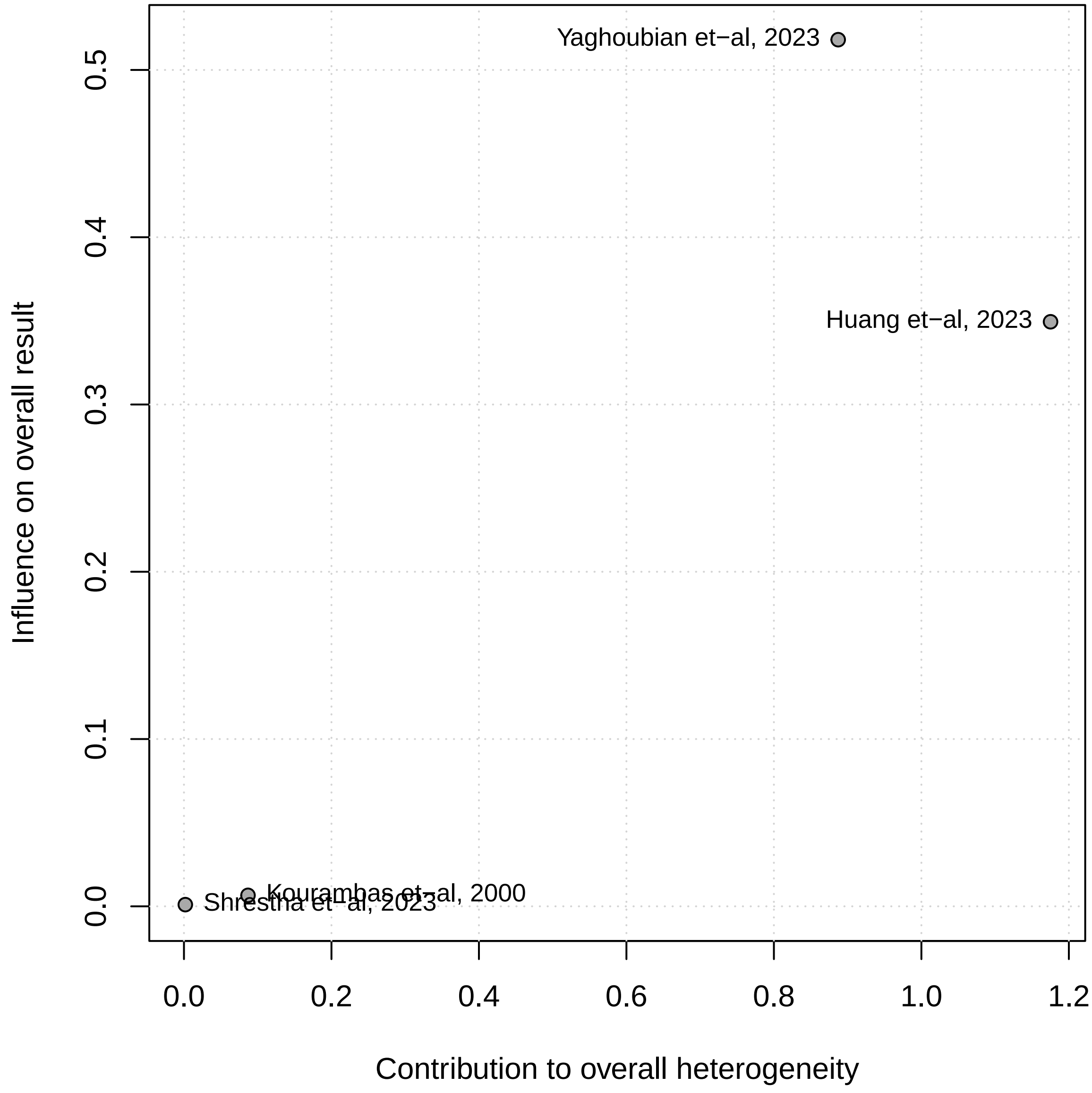

5.2.7 Leave One Out

```r
metainf(comp_insitu_rma)
```

```
## Influential analysis (common effect model)
##
##                                       RR           95%-CI p-value   tau^2
## Omitting Kourambas et-al, 2000    1.2804 [0.6234; 2.6300]  0.5009  0.0000
## Omitting Yaghoubian et-al, 2023   0.9080 [0.3862; 2.1346]  0.8249  0.0000
## Omitting Shrestha et-al, 2023     1.2246 [0.4915; 3.0510]  0.6636  0.3015
## Omitting Huang et-al, 2023        1.5817 [0.7112; 3.5177]  0.2609  0.0000
##
## Pooled estimate                   1.2429 [0.6170; 2.5037]  0.5429  0.0000
##                                      tau   I^2
## Omitting Kourambas et-al, 2000     0.0006  2.8%
## Omitting Yaghoubian et-al, 2023    0.0000  0.0%
## Omitting Shrestha et-al, 2023      0.5490  6.9%
## Omitting Huang et-al, 2023         0.0000  0.0%
##
## Pooled estimate                    0.0000  0.0%
##
## Details on meta-analytical method:
## - Mantel-Haenszel method
## - Restricted maximum-likelihood estimator for tau^2
```

5.3 Clavien Dindo III

5.3.1 Meta-analysis of Proportions for In Situ

```
insitu_data_com_III <- insitu_data_com %>% drop_na(in_situ_cd_III)
in_situ_comp_metaprop <- meta::metaprop(
  data = insitu_data_com_III,
  event = in_situ_cd_III,
  n = number_in_situ,
  studlab = paste(author, year, sep = ", "),
  method = "Inverse"
)
in_situ_comp_metaprop
```

```
## Number of studies: k = 4
## Number of observations: o = 173
## Number of events: e = 1
##
##                        proportion           95%-CI
## Common effect model        0.0145 [0.0042; 0.0487]
## Random effects model       0.0145 [0.0042; 0.0487]
##
## Quantifying heterogeneity:
##  tau^2 = 0; tau = 0; I^2 = 0.0% [0.0%; 84.7%]; H = 1.00 [1.00; 2.56]
##
## Test of heterogeneity:
##     Q d.f. p-value
##  0.10    3  0.9924
##
## Details on meta-analytical method:
## - Inverse variance method
## - Restricted maximum-likelihood estimator for tau^2
## - Logit transformation
## - Continuity correction of 0.5 in studies with zero cell frequencies
```

5.3.2 Meta-analysis of Proportions for Displacement

```
displacement_comp_metaprop <- metaprop(
  event = displacement_cd_III,
  n = number_displaced,
  data = insitu_data_com_III,
  studlab = paste(author, year, sep = ", "),
  method = "Inverse"
)
displacement_comp_metaprop
```

```
## Number of studies: k = 4
## Number of observations: o = 157
## Number of events: e = 4
##
##                        proportion           95%-CI
## Common effect model        0.0403 [0.0168; 0.0936]
## Random effects model       0.0378 [0.0142; 0.0968]
##
## Quantifying heterogeneity:
##  tau^2 = 0.1330 [0.0000; 8.0158]; tau = 0.3646 [0.0000; 2.8312]
##  I^2 = 0.0% [0.0%; 84.7%]; H = 1.00 [1.00; 2.56]
##
## Test of heterogeneity:
##     Q d.f. p-value
##  2.41    3  0.4918
##
## Details on meta-analytical method:
## - Inverse variance method
## - Restricted maximum-likelihood estimator for tau^2
## - Q-Profile method for confidence interval of tau^2 and tau
## - Logit transformation
## - Continuity correction of 0.5 in studies with zero cell frequencies
```

5.3.3 Meta-Analysis

```
comp_insitu_rma <- metabin(data = (insitu_data_com_III %>% subset(displacement_cd_III != 0)),
                    event.c = in_situ_cd_III,
                    n.c = number_in_situ,
                    event.e = displacement_cd_III,
                    n.e = number_displaced,
                    studlab = paste(author, year, sep = ", ")
                    )
comp_insitu_rma
```

```
## Number of studies: k = 2
## Number of observations: o = 228
## Number of events: e = 5
##
##                           RR            95%-CI    z p-value
## Common effect model  3.0000 [0.4805; 18.7320] 1.18  0.2398
## Random effects model 2.4854 [0.3339; 18.4984] 0.89  0.3740
##
## Quantifying heterogeneity:
##  tau^2 = 0; tau = 0; I^2 = 0.0%; H = 1.00
##
## Test of heterogeneity:
##     Q d.f. p-value
##  0.90    1  0.3431
##
## Details on meta-analytical method:
## - Mantel-Haenszel method
## - Inverse variance method
## - Restricted maximum-likelihood estimator for tau^2
## - Continuity correction of 0.5 in studies with zero cell frequencies
```

5.3.4 Forest plot

```r
forest(comp_insitu_rma,
       sortvar = TE)
```

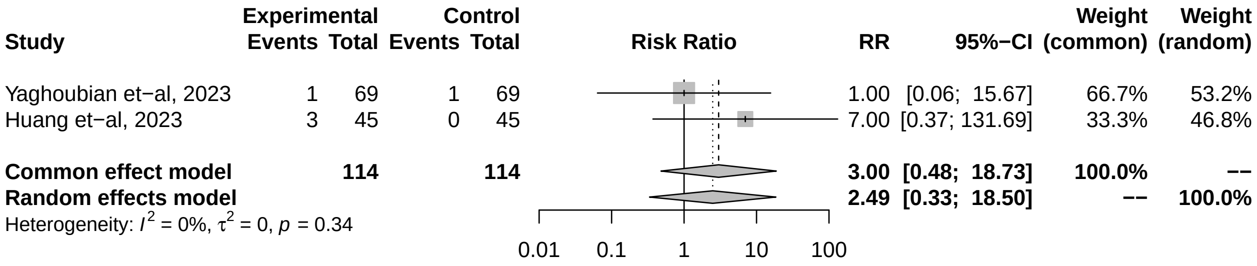

5.3.5 Trim and Fill

```r
trimfill(comp_insitu_rma)
```

```
## Warning in trimfill.meta(comp_insitu_rma): Minimal number of three studies for
## trim-and-fill method
```

```r
funnel((comp_insitu_rma))
```

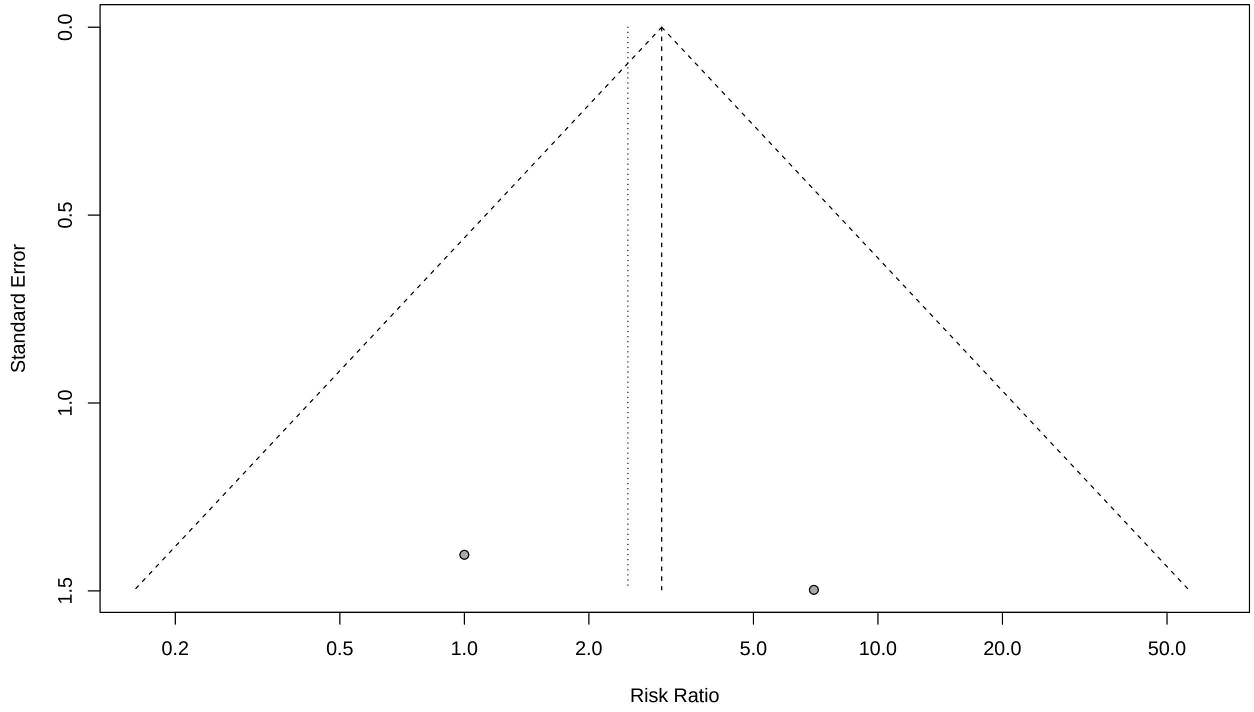


```r
forest((comp_insitu_rma), sortvar = TE)
```

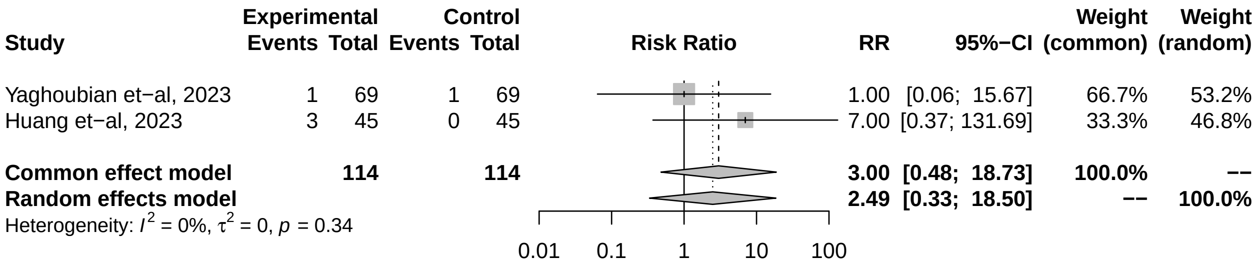

5.3.6 Baujat

```
baujat(comp_insitu_rma)
```

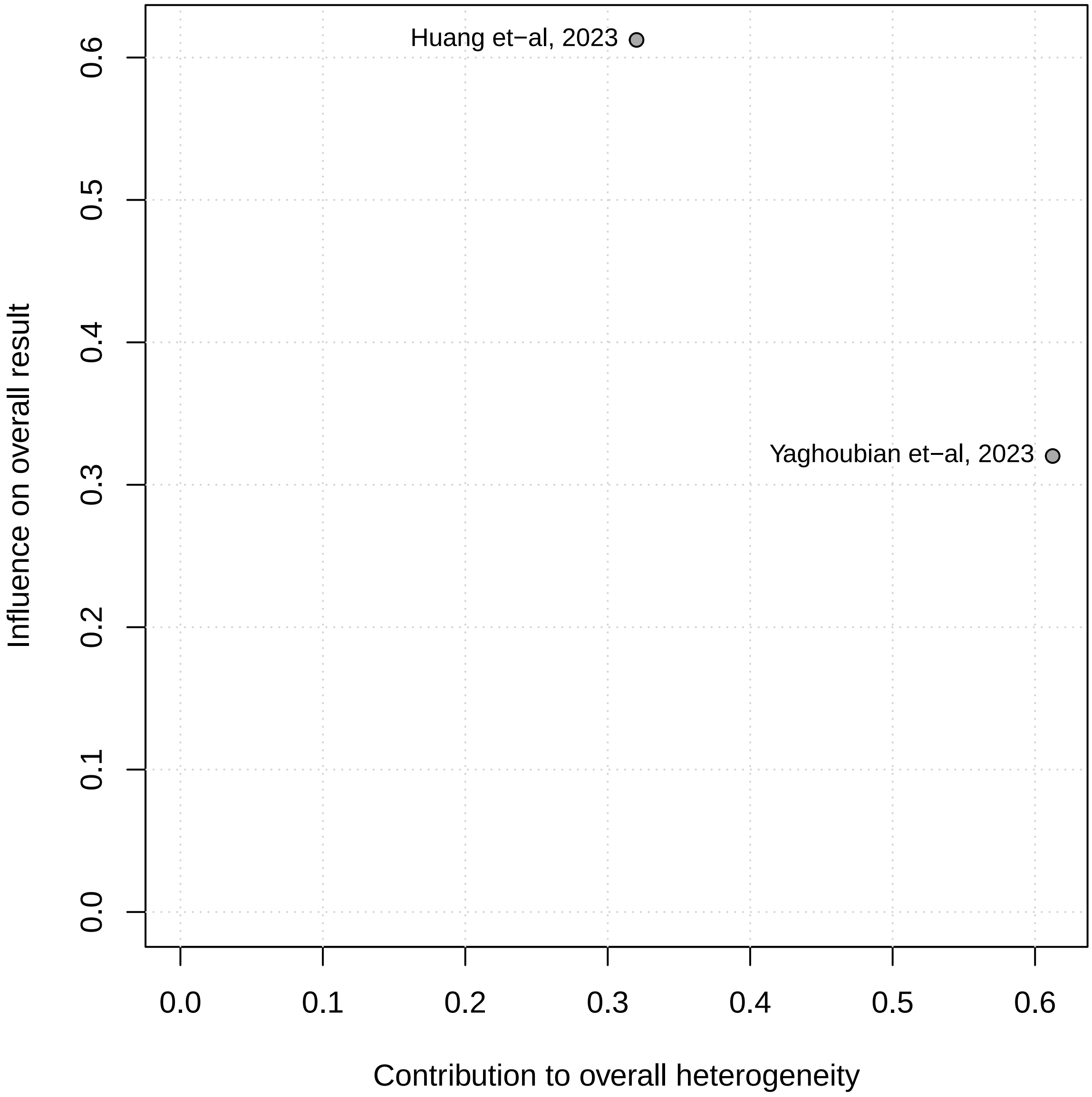

5.3.7 Leave One Out

```
metainf(comp_insitu_rma)
```

```
## Influential analysis (common effect model)
##
##                                           RR             95%-CI p-value   tau^2
## Omitting Yaghoubian et-al, 2023   7.0000 [0.3720; 131.7333]  0.1938
## Omitting Huang et-al, 2023        1.0000 [0.0638;  15.6684]  1.0000
##
## Pooled estimate                   3.0000 [0.4805;  18.7320]  0.2398  0.0000
##                                       tau   I^2
## Omitting Yaghoubian et-al, 2023
## Omitting Huang et-al, 2023
##
## Pooled estimate                    0.0000  0.0%
##
## Details on meta-analytical method:
## - Mantel-Haenszel method
## - Restricted maximum-likelihood estimator for tau^2
```

6 Operative time

6.1 Meta-analysis of individual components

6.1.1 In Situ

```
optime_data <- insitu_data %>% subset(select = c(author,
                                                 number_in_situ,
                                                 number_displaced,
                                  in_situ_operative_time,
                                  in_situ_operative_time_sd,
                                  displacement_operative_time,
                                  displacement_operative_time_sd))

optime_data %>% gt()
```

author	number_in_situ	number_displaced	in_situ_operative_time	in_situ_operative_time_sd	disp
Kourambas et-al	24	10	NA	NA	
Schuster et-al	59	19	64.0	NA	
Yaghoubian et-al	69	69	57.0	14.0	
Shrestha et-al	35	33	42.6	13.7	
Huang et-al	45	45	39.8	17.1	

```
metamean(data = optime_data,
         mean = in_situ_operative_time,
         sd = in_situ_operative_time_sd,
         n = number_in_situ)
```

```
## Number of studies: k = 3
## Number of observations: o = 232
##
##                            mean             95%-CI
## Common effect model  49.2990 [46.9436; 51.6544]
## Random effects model 46.5988 [36.0498; 57.1478]
##
## Quantifying heterogeneity:
##  tau^2 = 82.0322 [19.0886; >820.3218]; tau = 9.0572 [4.3690; >28.6413]
##  I^2 = 95.4% [89.7%; 97.9%]; H = 4.64 [3.12; 6.92]
##
## Test of heterogeneity:
##      Q d.f.  p-value
##  43.13    2 < 0.0001
##
## Details on meta-analytical method:
## - Inverse variance method
## - Restricted maximum-likelihood estimator for tau^2
## - Q-Profile method for confidence interval of tau^2 and tau
## - Untransformed (raw) means
```

6.1.2 Displacement

```
metamean(data = optime_data,
         mean = displacement_operative_time,
         sd = displacement_operative_time_sd,
         n = number_displaced)
```

```
## Number of studies: k = 3
## Number of observations: o = 176
##
##                          mean             95%-CI
## Common effect model  59.9583 [57.9791; 61.9374]
## Random effects model 51.3082 [35.7012; 66.9151]
##
## Quantifying heterogeneity:
##  tau^2 = 184.2166 [45.8451; >1842.1657]; tau = 13.5726 [6.7709; >42.9205]
##  I^2 = 97.8% [95.8%; 98.8%]; H = 6.72 [4.88; 9.24]
##
## Test of heterogeneity:
##      Q d.f.  p-value
##  90.20    2 < 0.0001
##
## Details on meta-analytical method:
## - Inverse variance method
## - Restricted maximum-likelihood estimator for tau^2
## - Q-Profile method for confidence interval of tau^2 and tau
## - Untransformed (raw) means
```

6.2 Meta-Analysis

6.2.1 Result

```
op_time_insitu_rma <- metacont(data = insitu_data,
                    mean.c = in_situ_operative_time,
                    sd.c = in_situ_operative_time_sd,
                    n.c = number_in_situ,
                    mean.e = displacement_operative_time,
                    sd.e = displacement_operative_time_sd,
                    n.e = number_displaced,
                    studlab = paste(author, year, sep = ", ")
                    )
op_time_insitu_rma
```

```
## Number of studies: k = 3
## Number of observations: o = 408
##
##                           MD             95%-CI    z  p-value
## Common effect model  6.7738 [3.6161;  9.9314] 4.20 < 0.0001
## Random effects model 5.6165 [0.4008; 10.8323] 2.11   0.0348
##
## Quantifying heterogeneity:
##  tau^2 = 11.7129 [0.0000; >117.1293]; tau = 3.4224 [0.0000; >10.8226]
##  I^2 = 55.0% [0.0%; 87.1%]; H = 1.49 [1.00; 2.79]
##
## Test of heterogeneity:
##     Q d.f. p-value
##  4.44    2  0.1086
##
## Details on meta-analytical method:
## - Inverse variance method
## - Restricted maximum-likelihood estimator for tau^2
## - Q-Profile method for confidence interval of tau^2 and tau
```

6.2.2 Forest plot

```r
forest(op_time_insitu_rma,
       sortvar = TE)
```

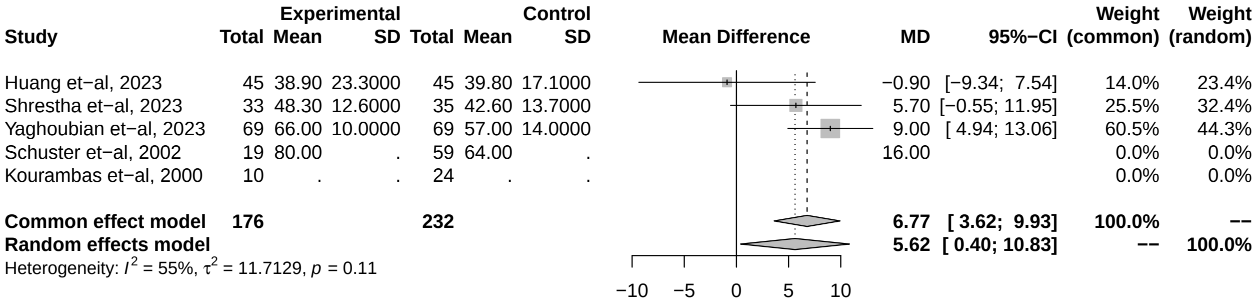

6.2.3 Trim and Fill

```r
trimfill(op_time_insitu_rma)
```

```
## Warning in trimfill.meta(op_time_insitu_rma): 2 observation(s) dropped due to
## missing values

## Number of studies: k = 5 (with 2 added studies)
## Number of observations: o = 454
##
##                          MD            95%-CI    z p-value
## Random effects model 9.0000 [3.3080; 14.6920] 3.10  0.0019
##
## Quantifying heterogeneity:
##  tau^2 = 30.5400 [2.2979; >305.3996]; tau = 5.5263 [1.5159; >17.4757]
##  I^2 = 68.5% [18.9%; 87.8%]; H = 1.78 [1.11; 2.86]
##
## Test of heterogeneity:
##      Q d.f. p-value
##  12.70    4  0.0128
##
## Details on meta-analytical method:
## - Inverse variance method
## - Restricted maximum-likelihood estimator for tau^2
## - Q-Profile method for confidence interval of tau^2 and tau
## - Trim-and-fill method to adjust for funnel plot asymmetry (L-estimator)
```

```r
funnel((op_time_insitu_rma))
```

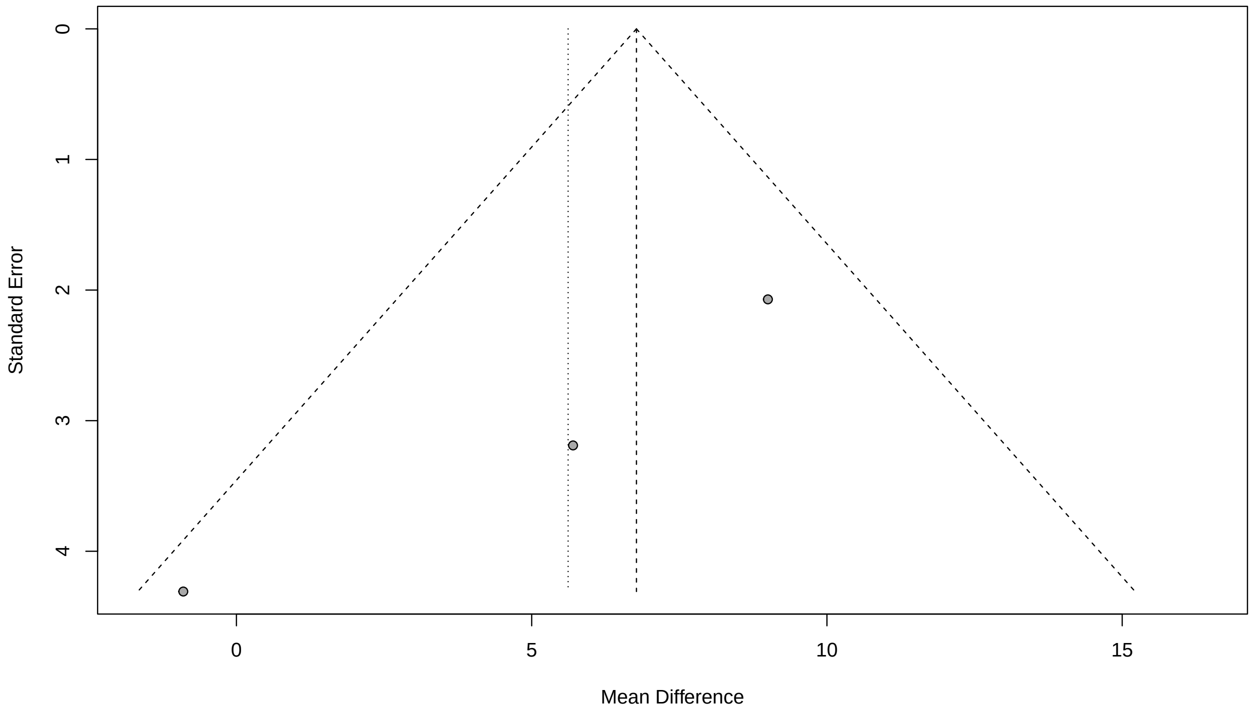


```r
forest((op_time_insitu_rma), sortvar = TE)
```

Study	Experimental Total	Experimental Mean	Experimental SD	Control Total	Control Mean	Control SD	Mean Difference	MD	95%−CI	Weight (common)	Weight (random)
Huang et−al, 2023	45	38.90	23.3000	45	39.80	17.1000		−0.90	[−9.34; 7.54]	14.0%	23.4%
Shrestha et−al, 2023	33	48.30	12.6000	35	42.60	13.7000		5.70	[−0.55; 11.95]	25.5%	32.4%
Yaghoubian et−al, 2023	69	66.00	10.0000	69	57.00	14.0000		9.00	[4.94; 13.06]	60.5%	44.3%
Schuster et−al, 2002	19	80.00	.	59	64.00	.		16.00		0.0%	0.0%
Kourambas et−al, 2000	10	.	.	24	.	.				0.0%	0.0%
**Common effect model**	**176**			**232**				**6.77**	**[3.62; 9.93]**	**100.0%**	**−−**
**Random effects model**								**5.62**	**[0.40; 10.83]**	**−−**	**100.0%**

Heterogeneity: $I^2 = 55\%$, $\tau^2 = 11.7129$, $p = 0.11$

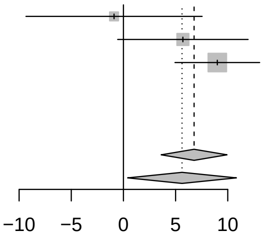

6.2.4 Baujat

```
#baujat(op_time_insitu_rma)
```

7 Overall Results

7.0.1 In Situ vs control

```
overall_results <- cbind(
  "Outcome" = c(
    "Stone Free Rate",
    "Stone Free Rate (RCT only)",
    "Stone Free Rate (No Fragments)",
    "Stone Free Rate (10-20mm only)",
    "Post-Operative Stent Rate",
    "Complication Rate",
    "Operative Time"
  ),
  "papers_n" = c(
    sf_insitu_rma$k,
    sf_insitu_rct_rma$k,
    sf_definition_data_rma$k,
    stones_10_20_data_rma$k,
    stent_insitu_rma$k,
    comp_insitu_rma$k,
    op_time_insitu_rma$k
  ),
  "MA_Result" = c(
    sf_insitu_rma$TE.fixed %>% exp(),
    sf_insitu_rct_rma$TE.fixed %>% exp(),
    sf_definition_data_rma$TE.fixed %>% exp(),
    stones_10_20_data_rma$TE.fixed %>% exp(),
    stent_insitu_rma$TE.fixed %>% exp(),
    comp_insitu_rma$TE.random %>% exp(),
    op_time_insitu_rma$TE.random
  ),
  "Lower_CI" = c(
    sf_insitu_rma$lower.fixed %>% exp(),
    sf_insitu_rct_rma$lower.fixed %>% exp(),
    sf_definition_data_rma$lower.fixed %>% exp(),
    stones_10_20_data_rma$lower.fixed %>% exp(),
    stent_insitu_rma$lower.fixed %>% exp(),
    comp_insitu_rma$lower.random %>% exp(),
    op_time_insitu_rma$lower.random
  ),
  "Upper_CI" = c(
    sf_insitu_rma$upper.fixed %>% exp(),
    sf_insitu_rct_rma$upper.fixed %>% exp(),
    sf_definition_data_rma$upper.fixed %>% exp(),
    stones_10_20_data_rma$upper.fixed %>% exp(),
    stent_insitu_rma$upper.fixed %>% exp(),
    comp_insitu_rma$upper.random %>% exp(),
    op_time_insitu_rma$upper.random
  ),
  "P" = c(
    sf_insitu_rma$pval.fixed,
    sf_insitu_rma$pval.fixed,
    sf_definition_data_rma$pval.fixed,
```

```
    stones_10_20_data_rma$pval.fixed,
    stent_insitu_rma$pval.fixed,
    comp_insitu_rma$pval.random,
    op_time_insitu_rma$pval.random
  ),
  "Model" = c("Fixed", "Fixed", "Fixed", "Fixed", "Fixed", "Random", "Random")
) %>% as_tibble()

overall_results$MA_Result <-
  round(as.numeric(overall_results$MA_Result), digits = 2)
overall_results$Lower_CI <-
  round(as.numeric(overall_results$Lower_CI), digits = 2)
overall_results$Upper_CI <-
  round(as.numeric(overall_results$Upper_CI), digits = 2)
overall_results$P <-
  round(as.numeric(overall_results$P), digits = 2)
overall_results$P <-
  ifelse(overall_results$P < 0.001, "<0.001", overall_results$P)

overall_results %>% gt() %>% cols_merge(columns = c(Lower_CI, Upper_CI),
                                        pattern = "({1}-{2})") %>% cols_merge(columns = c(MA_Result, Lo
```

Outcome	Papers, n	MA Result (95% CI)	P	Model
Stone Free Rate	5	1.21 (1.10-1.34)	<0.001	Fixed
Stone Free Rate (RCT only)	3	1.21 (1.07-1.36)	<0.001	Fixed
Stone Free Rate (No Fragments)	3	1.23 (1.09-1.38)	<0.001	Fixed
Stone Free Rate (10-20mm only)	4	1.18 (1.05-1.32)	<0.001	Fixed
Post-Operative Stent Rate	5	0.98 (0.86-1.11)	0.71	Fixed
Complication Rate	2	2.49 (0.33-18.50)	0.37	Random
Operative Time	3	5.62 (0.40-10.83)	0.03	Random

7.1 Summary Forest Plot

```
overall_binary <-
  overall_results %>% subset(Outcome != "Operative Time")

binary_plot <- overall_binary %>%
  forestplot(
    mean = MA_Result,
    lower = Lower_CI,
    upper = Upper_CI,
    zero = 1,
    labeltext = c(Outcome, papers_n, MA_Result, Lower_CI, Upper_CI, P),
  vertices = TRUE,
  title = "Forest plot of MA Outcomes for Binary Outcomes",
  clip = c(-1, 2.5),
  xlab = "RR [95% CI]",
  graph.pos = 3
) %>% fp_set_style(
    box = c("black"),
    line = "black",
    txt_gp = fpTxtGp(
      ticks = gpar(fontfamily = "", cex = 1),
      xlab  = gpar(fontfamily = "", cex = 1)
    )) %>% fp_add_lines("steelblue") %>%
  fp_add_header("Outcome",
                "Studies, n",
                  "RR",
                "[ 95%", " CI ]",
                "P-Value")  %>% fp_decorate_graph(box = TRUE) %>% fp_set_zebra_style("#EFEFEF")
binary_plot
```

Forest plot of MA Outcomes for Binary Outcomes

Outcome	Studies, n	RR	[95%	CI]	P−Value
Stone Free Rate	5	1.21	1.1	1.34	<0.001
Stone Free Rate (RCT only)	3	1.21	1.07	1.36	<0.001
Stone Free Rate (No Fragments)	3	1.23	1.09	1.38	<0.001
Stone Free Rate (10−20mm only)	4	1.18	1.05	1.32	<0.001
Post−Operative Stent Rate	5	0.98	0.86	1.11	0.71
Complication Rate	2	2.49	0.33	18.5	0.37

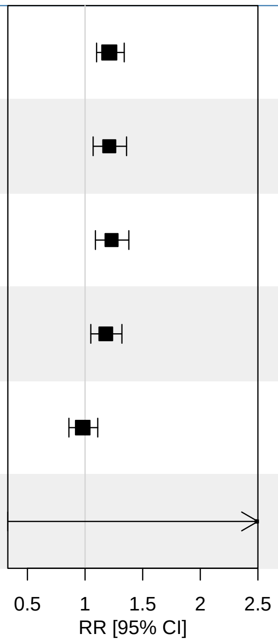

Supplement: Supplementary file 2 — Appendix S2. In situ vs displacement. [file BJU-135-399-s002.pdf]
