# Supplementary material for: Ureteroscopy and lasertripsy for lower pole stones <2 cm, in situ vs displacement? A systematic review and meta‐analysis
Source: BJU Int. 2024 Oct 13;135(3):399–407. doi: 10.1111/bju.16534 (PMC11842885; doi:10.1111/bju.16534)
Supplement: Supplementary file 3 — Figure S1. Forest plot summarising the primary outcome of SFRs of in situ (control group) vs displacement (experimental group) in the management of lower pole stones. ‘Events’ represent number of stone‐free patients in each group. [file BJU-135-399-s003.docx]

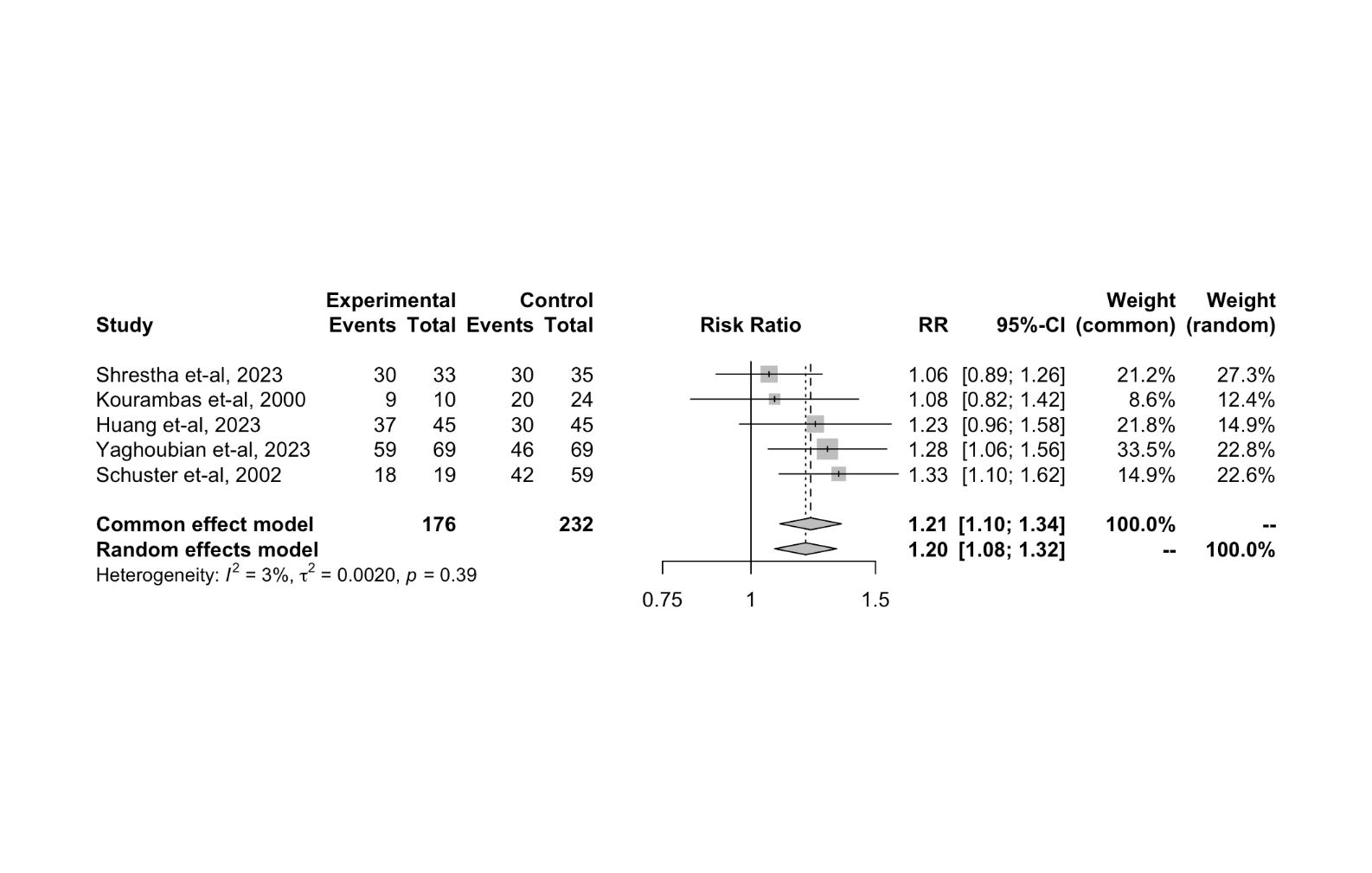


*Supplementary Figure 1: Forest plot summarising the primary outcome of stone free rates of in-situ (control group) versus displacement (experimental group) in the management of lower-pole stones. “Events” represent number of stone free patients in each group*
