# Supplementary material for: Ureteroscopy and lasertripsy for lower pole stones <2 cm, in situ vs displacement? A systematic review and meta‐analysis
Source: BJU Int. 2024 Oct 13;135(3):399–407. doi: 10.1111/bju.16534 (PMC11842885; doi:10.1111/bju.16534)
Supplement: Supplementary file 4 — Figure S2. Funnel plot demonstrating trim‐and‐fill analysis for meta‐analysis of SFRs. [file BJU-135-399-s004.docx]

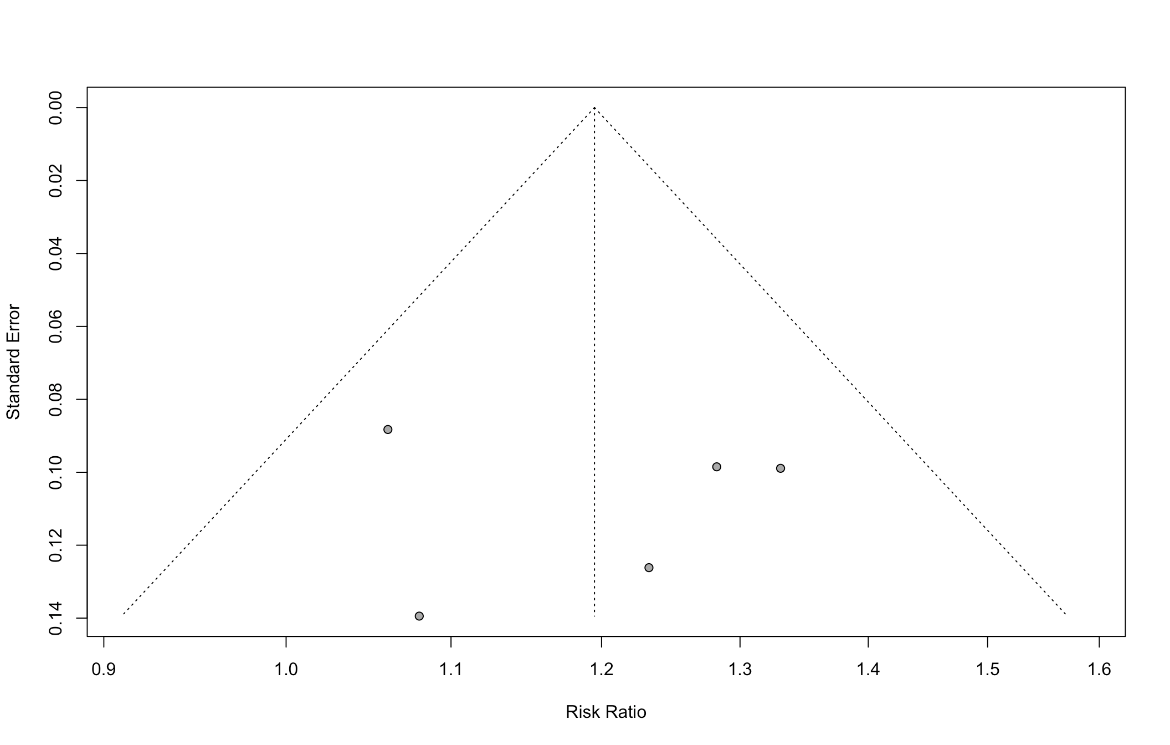


*Supplementary Figure 2: Funnel plot demonstrating trim and fill analysis for meta analysis of stone-free rates*
