# Supplementary material for: Ureteroscopy and lasertripsy for lower pole stones <2 cm, in situ vs displacement? A systematic review and meta‐analysis
Source: BJU Int. 2024 Oct 13;135(3):399–407. doi: 10.1111/bju.16534 (PMC11842885; doi:10.1111/bju.16534)
Supplement: Supplementary file 5 — Figure S3. Forest plot summarising the secondary outcome of perioperative stenting between in situ (control group) vs displacement (experimental group). [file BJU-135-399-s007.docx]

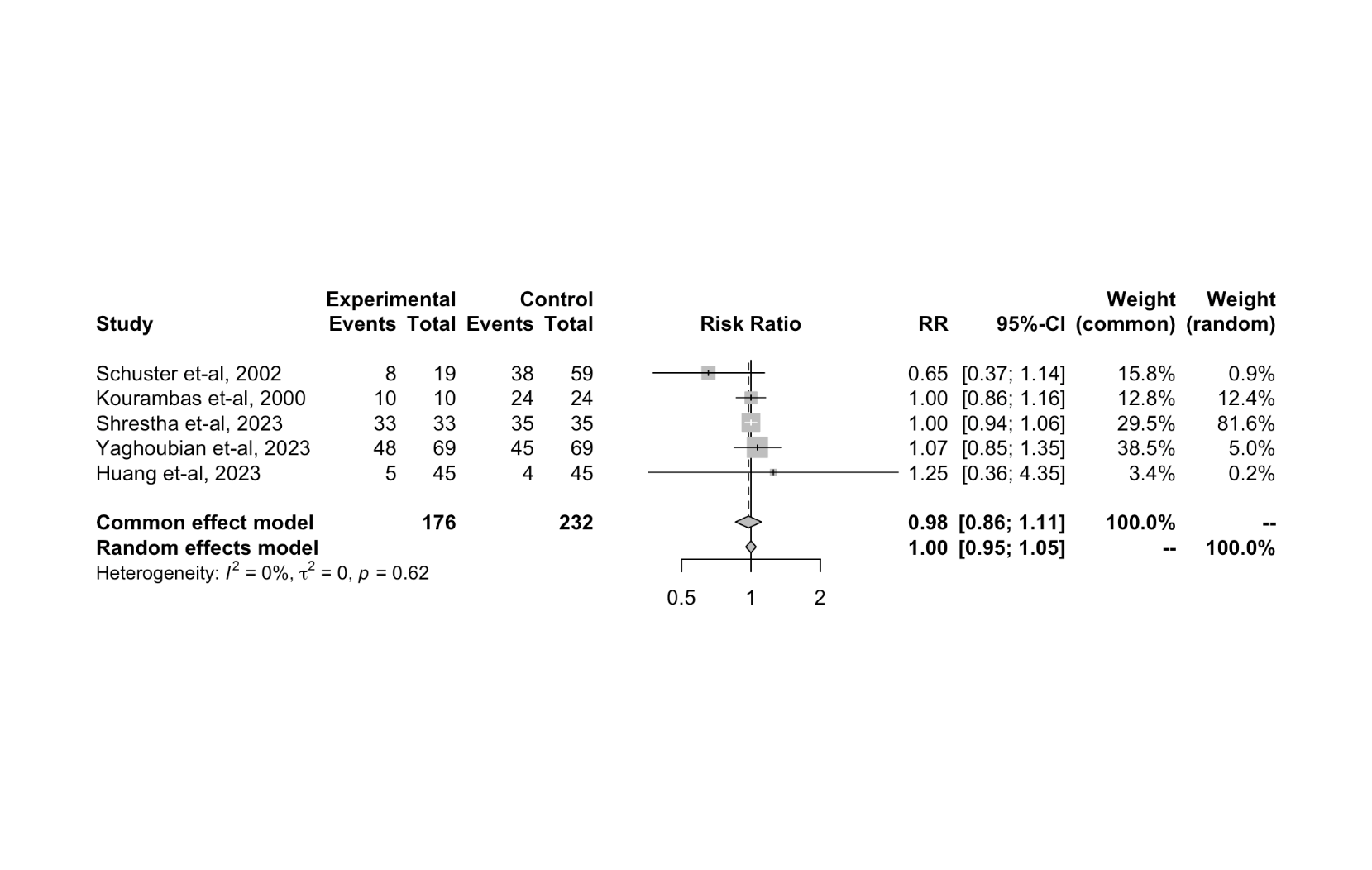


*Supplementary Figure 3: : Forest plot summarising the secondary outcome of peri-operative stenting between in-situ (control group) versus displacement (experimental group).*
