# Supplementary material for: Ureteroscopy and lasertripsy for lower pole stones <2 cm, in situ vs displacement? A systematic review and meta‐analysis
Source: BJU Int. 2024 Oct 13;135(3):399–407. doi: 10.1111/bju.16534 (PMC11842885; doi:10.1111/bju.16534)
Supplement: Supplementary file 6 — Figure S4. Forest plot summarising the secondary outcome of complication rates between in situ (control group) vs displacement (experimental group). [file BJU-135-399-s001.docx]

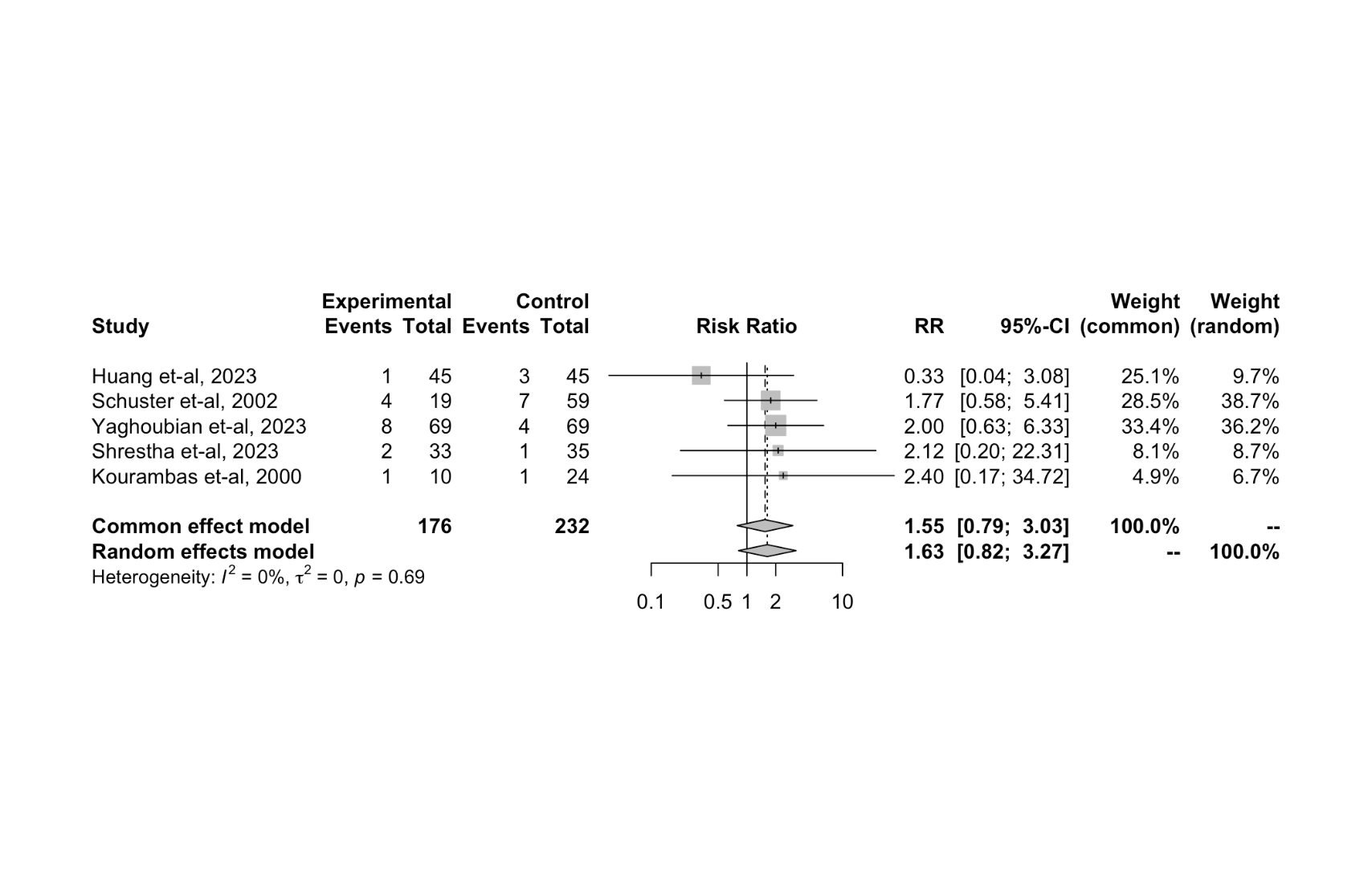


*Supplementary Figure 4: : Forest plot summarising the secondary outcome of complication rates between in-situ (control group) versus displacement (experimental group).*
