# Supplementary material for: Ureteroscopy and lasertripsy for lower pole stones <2 cm, in situ vs displacement? A systematic review and meta‐analysis
Source: BJU Int. 2024 Oct 13;135(3):399–407. doi: 10.1111/bju.16534 (PMC11842885; doi:10.1111/bju.16534)
Supplement: Supplementary file 7 — Figure S5. Baujat plot of primary outcome—SFRs—demonstrating each studies contribution to heterogeneity. [file BJU-135-399-s005.docx]

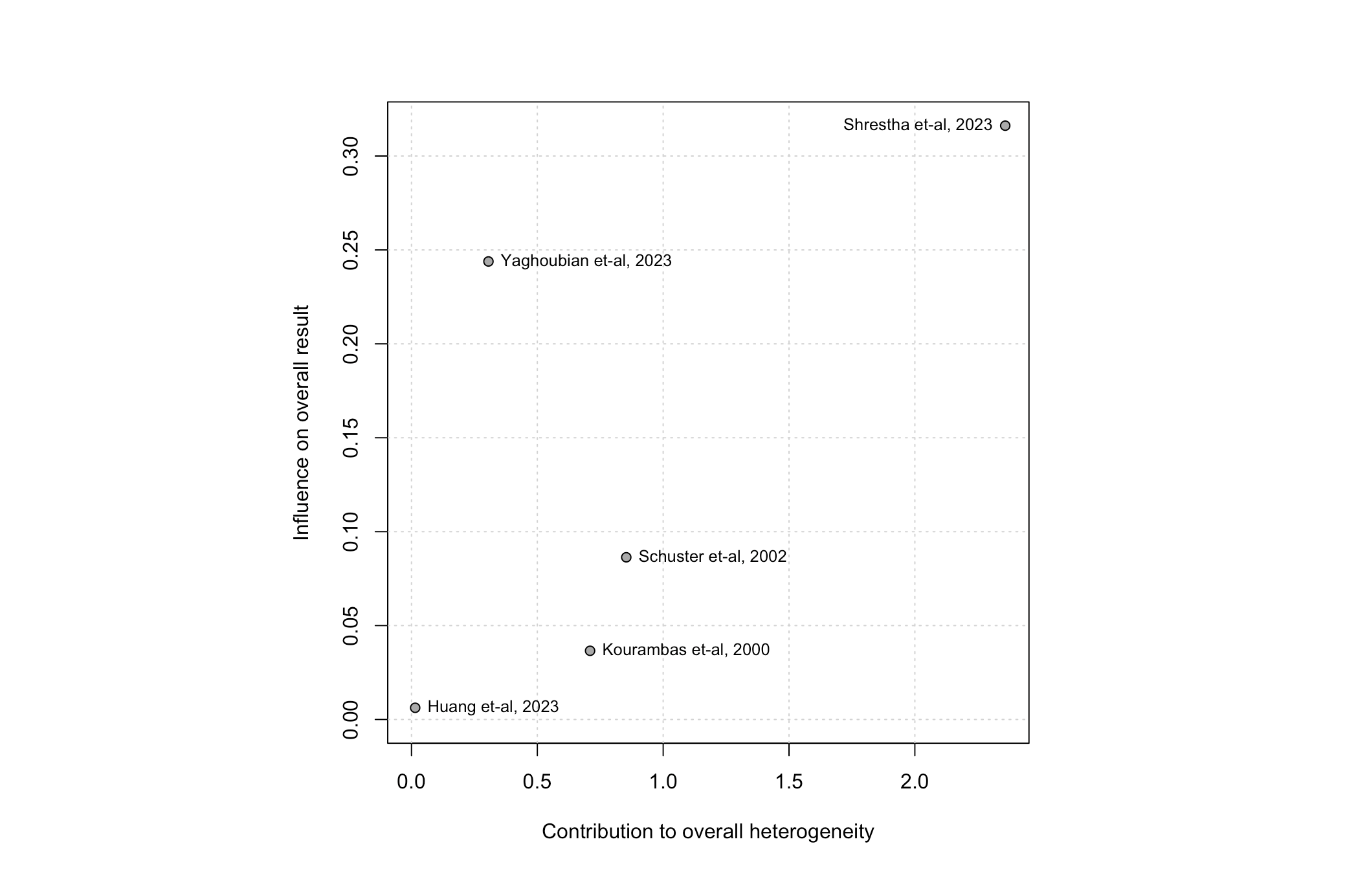


*Supplementary Figure 5: : Baujat plot of primary outcome – stone free rates – demonstrating each studies contribution to heterogenicity*
